# A Mathematical Modeling of Freezing Process in the Batch Production of Ice Cream

**DOI:** 10.3390/foods10020334

**Published:** 2021-02-04

**Authors:** Paolo Giudici, Antonietta Baiano, Paola Chiari, Luciana De Vero, Babak Ghanbarzadeh, Pasquale Massimiliano Falcone

**Affiliations:** 1 Department of Life Sciences, University of Modena and Reggio Emilia, 42122 Reggio Emilia, Italy; pavel.albiond@gmail.com (P.G.); paola.chiari.consulting@gmail.com (P.C.); luciana.devero@unimore.it (L.D.V.); 2 Dipartimento di Scienze Agrarie, Alimenti, Risorse Naturali e Ingegneria, University of Foggia, 71122 Foggia, Italy; antonietta.baiano@unifg.it; 3 Department of Food Science and Technology, University of Tabriz, Tabriz 51666-16471, Iran; ghanbarzadeh@tabrizu.ac.ir; 4 Department of Agricultural, Food and Environmental Sciences, Marche University Polytechnical, 60131 Ancona, Italy

**Keywords:** Gompertz’s function, ice cream, initial freezing point, supercooling, time-temperature profile

## Abstract

This study deals with the mathematical modeling of crystallization kinetics occurring during batch production of the ice cream. The temperature decrease was recorded in-situ through a computerized wireless system. A robust pattern-recognition algorithm of the experimental cooling curves was developed to determine the initial freezing point. The theoretical freezing point was used to calibrate the whole time-temperature profile. Finally, a modified Gompertz’s function was used to describe the main steps of crystallization kinetics. Derivative analysis of the Gompertz’s function allowed to determine the time-temperature physical markers of dynamic nucleation, ice crystal growth and air whipping. Composition and freezing properties were used as input variables in multivariate analysis to classification purposes of the ice cream mixtures as a function of their ability to produce high-quality ice cream. The numerical analysis of the whole cooling curve was used to build predictive models of the ice cream quality indices.

## 1. Introduction

Ice cream is the most widely consumed frozen dairy dessert, with a global market value of $68,072 million in 2016 [1] The meaning of the term “ice cream” varies from country to country, due to different regulations and traditions and, thus, covers a wide range of ingredients and composition, as well as product texture and manufacturing processes. In addition, in the last decades, there is an increasing interest to produce nourishing and healthy ice cream as novelty products by adding fruits, spices, protein-rich constituents, probiotics and prebiotics. Several studies were performed to develop new formulations, new structures and to add health properties to ice cream products, including, for instance, selected lactic acid bacteria with functional effects [2,3].

From a manufacturing point of view, ice cream can be categorized as industrial and artisanal ice cream as a function of the process technologies and production scale. The artisanal ice cream production involves the following operations: preparation of a liquid mixture generally containing ingredients such as milk fat, milk-solids-not-fat (the principal source of protein), sugars (or sweeteners), hydrocolloids (stabilizers and emulsifiers); aging of this mixture; simultaneous whipping and freezing under high-shear dynamic conditions; incorporation of flavoring ingredients to this partially frozen mixture; tempering, shaping and serving of the final product. In the case of the industrial production, another freezing step under static conditions is required after shaping. The first freezing step is carried out using scraped-surface freezers, to decrease the mixture temperature below its freezing point (typically at −5 °C) to obtain a soft, semi-frozen texture. In the artisanal process, scraped-surface freezers are batch plants able to incorporate air under atmospheric conditions (overrun below 40%). Instead, in the industrial process, scraped-surface freezers are continuous plants able to incorporate air under the pressure to reach an overrun of 100%. The second freezing step required in the industrial production is carried out using forced-air convection freezers to reduce the internal temperature to −25/−30 °C (below its glass transition) thus resulting in a harder texture and a longer shelf-life. 

From the qualitative point of view, industrial and artisanal ice creams mainly differ for air content and texture properties [4]. The liquid mixtures used to produce the industrial ice cream contain total solids up to 42% by weight and consist of milk fat, milk-solid-not-fat, sweeteners and hydrocolloids (stabilizers and emulsifiers) up to 18%, 8%, 17%, 0.5% by weight, respectively [5]. Their relatively high-fat and hydrocolloid contents together with the low water content are required to decrease the interfacial properties of the liquid mixtures and, therefore, their ability to hold a higher amount of air in the final product. At serving temperatures, the industrial ice cream appears very stiff, icy and greatly stable. The ingredients traditionally used to produce artisanal ice creams (the Italian-style gelato is an example of this category) are represented by milk fat, milk-solid-not-fat, sweeteners and hydrocolloids up to 8%, 7.5%, 16%, 0.2% by weight, respectively, to reach a total solid content up to 32% by weight [5]. The artisanal ice cream is served at −13 °C/−15 °C resulting in a scoopable and easy-to-melt texture. 

Under a microstructural point of view, both industrial and artisanal ice creams can be considered three-phase colloidal systems consisting of ice crystals; unfrozen serum phase, that in turn contains clusters of partially crystalline and partially coalesced fat globules, all interacting with hydrocolloids to create a three-dimensional viscoelastic network; air cells, entrapped into the serum phase [6,7,8,9,10]. The two key operations concurring to the development of the ice cream microstructure are the aging of the starting liquid mixture and the dynamic freezing performed under shear. Usually, the composition of the starting mixtures and the aging conditions are empirically selected to improve the final physical properties (which include colloidal/emulsion stability, density, viscosity, interfacial and surface tension, specific heat and freezing point), the cooling behavior during the first freezing step (extent of supercooling, ice nucleation, ice growth/ripening and air whipping) and, finally, the microstructure of the final product. The aging is usually carried out at −4 °C for a time ranging from 4 to 24 h. Under refrigerated aging, proteins increase their hydration state and milk fat globules undergo nearly complete crystallizing with a rate that depends on the type of fats and emulsifiers used. More specifically, the emulsifiers displace the hydrated proteins at the fat globule interface to provide significant increases in viscosity. The sweeteners used in the formulation of the ice cream mixture play a major role in determining the initial freezing point [11]. 

Sensory quality and melting resistance are two key quality aspects of the ice cream and they are strictly dependent on what takes place in the barrel of the scraped-surface heat exchanger (SSHE) used to produce the ice cream, which is the place in which ice nucleation starts. Many tiny ice crystal nuclei are formed, scraped and melt into the bulk of the ice cream mixture where the larger-size crystals grown. Once the product leaves the SSHE, no further nucleation takes place but the existing crystals grow with time and such phenomenon is accelerated under temperature variation. Ice crystals larger than 40–50 μm result in coarse or grainy texture if present in sufficient number. Both the rate and extent of water crystallization affect the amount and the size distribution of the ice crystals in the ice cream. Water crystallization that takes place under dynamic conditions inside the SSHE is the main cause of the formation of a viscoelastic network inside the unfrozen phase, which in turn limits the ice crystal growth and the stable incorporation of air in the final product. Water crystallization causes the increase of both solute concentration and viscosity of the unfrozen phase remaining at the end of the cooling process. Instead, mechanical shearing causes structure transition from slurry ice cream mixture to soft-solid ice cream by promoting shear-induced destabilization of the milk fat globules and the stable incorporation of finely dispersed air cells (overrun). The increasing of the overall stiffness of the ice cream mixture during freezing is due to the intermolecular interactions occurring under high-shear rate among fats, proteins and hydrocolloids from colloidal to the macroscopic scale.

Thus, the quantitative description of the water crystallization kinetics is of fundamental importance to understand and manage the ice cream production but it is difficult to describe by mechanistic models. Currently, there are several gaps in both scientific and technical knowledges on the topic. From the scientific point of view, there is a gap between the theoretical knowledge based on the thermodynamics of water crystallization (well established within ideal liquid solutions for which both the Clausius-Clapeyron’s and Raoult’s laws can apply satisfactorily) and the knowledge about this phase transition within heterogeneous and high-concentrated liquid systems, such as the ice cream formulations. The partial freezing of ice cream mixture occurring under dynamic conditions involves complex thermodynamic and kinetic mechanisms such as heat and mass transfer, supercooling, heterogeneous nucleation, as well as changes in thermo physical properties, surface curvature effects and so forth. Furthermore, multiple re-crystallization/melting and air incorporation must be considered. The equilibrium temperature at which the water crystallization takes place in the ice cream mixtures strictly depends on the ability of colligative solutes to depress the freezing point. Initial freezing point and freezable water fraction are the two parameters required to determine the temperature dependence of the frozen water fraction. The importance of freezing point depression in the ice cream production has induced researchers to develop models based on mix composition. As recently reviewed [5], some empirical models available in literature are based on measurements performed on diluted mix and the results are extrapolated aiming to be applied on the undiluted system. Other models are based on the freezing point depression of pure sucrose solutions and the estimated “sucrose equivalency” of various sweeteners and milk salts with respect to sucrose. Under the industrial standpoint, batch ice cream production is characterized by a low level of innovation, mainly due to the low level of automatic technologies available to control both the freezing and whipping operations. The mechanical characteristics of the scraped-surface freezers, which include shape and number of scraping blades and their rotational speed, also play a role in the structure formation, determining the spatial distribution of shear stress and freezing temperature inside the freezer barrel. It is widely recognized that ice nucleation and ice growth occur at different rates as a function of the axial and radial distance from freezing surface: ice nucleation quickly occurs on the cold surface of the freezer, while ice growth occurs faster inside the bulk [12,13,14,15,16]. In batch production the final quality of the ice cream is usually obtained with low throughput and high variability. Concerning managing of freezing operation, the highest level of controlling technology available among the commercial SSHE freezers used in batch ice cream production is represented by programmable automatic shutoff systems based on the rough ability to measure the desired stiffness in the final product. Stiffness is monitored by registering the increasing percentage of dasher motor load during freezing: when it achieves the desired set point, then the refrigeration system is automatically shut off, while the scraping blades continue to whip the product. Stiffness is a coarse parameter able to inform the producer only about the overall resistance encountered by the rotating blades. Stiffness is a bulk rheological property: different average sizes and size distributions of the ice crystals as well as of the air cells can result in the same overall stiffness but in different final texture and melting resistance. The traditional approach to manage freezing operations in batch ice cream production is based on the operator experience and in the trial-and-error assessment for setting ice cream formulations and freezing time. The time to shut off the refrigeration system is usually determined by the operator by drawing a small portion of ice cream and checking temperature. When the ice cream is drawn from the freezer, it should be stiff enough to form a “ribbon”, yet soft enough to slowly settle into the container and to lose its shape slowly.

The technological aim of freezing in the SSHE is to obtain the rapid formation of small ice crystals and to develop the desired overrun. The drawing temperature of ice cream (−5 to −7 °C) from the batch freezer is slightly higher than that used for continuous freezers and residence times in the freezer barrel are longer for batch processing (5–6 min). This results in larger ice crystals. Furthermore, the overrun control in the batch freezer is difficult because the scraping blades continue to operate during unloading of the freezer and the air is present at atmospheric pressure. It is useful point out that subjective evaluation of the time to shut off the refrigeration system as determined by the operator may produce high variability both in the size of the ice crystals and in the product yield (overrun). By prolonging the freezing time beyond the set point of desired stiffness, the amount of the incorporated air reaches a maximum and then decreases as a balance between the rheological, interfacial properties and mechanical stress established inside the freezing mixture [9,10,17,18]; at the meantime, smaller crystals melt determining the increase of the bigger ones [5], because of the rising and lowering in temperature as electronically modulated to keep constant the desired stiffness in the ice cream mixture before the exit from the SSHE. For this reason, it is important to empty the freezer quickly. Shutting off the refrigeration too soon can result in (i) extended whipping time with possible failure to obtain desired overrun, (ii) smoother texture provided the product does not have to warm excessively to produce desired overrun and (iii) coarse texture if product warms excessively to produce desired overrun. Likewise, failure to turn off the refrigeration soon enough can result in (i) stiffer ice cream with a lower temperature, (ii) extended whipping time to obtain desired overrun and (iii) inability to remove the entire product from the barrel. 

The above considerations highlight the need to identify, from one hand, the optimal technological properties of the starting ice cream mixture and from another hand, an objective strategy to manage the freezing/whipping operations in batch ice cream production. Hard work is required to realize simultaneously the two key technological properties, that is, the ability to entrap the finely dispersed air cells (referred as to ”whippability”) and the ability to support fast crystallization of the water molecules aiming to obtain finely dispersed ice crystals. Both these properties depend on the composition of the starting formulations, characteristics of the freezer and desired quality of the ice cream. To optimize the air incorporation and prevent the excessive growth of the ice crystals, an optimal composition of the starting liquid mixture must be considered a technological pre-requisite for ice cream production. The present work provides a new strategy to evaluate in-situ the optimal technological properties under batch freezing conditions. In particular, this paper suggests a suitable setup for in-bulk temperature measurement inside the barrel of the SSHE for batch production of ice cream and describes a numerical approach to analyze the time-temperature history (i.e., the whole cooling curve) experienced by the ice cream mixture, aiming to characterize the kinetics of water crystallization and air incorporation. The most significant kinetic properties were correlated to the overrun, melting and sensory properties providing predictive statistic models.

## 2. Materials and Methods

**Scheme 1:**
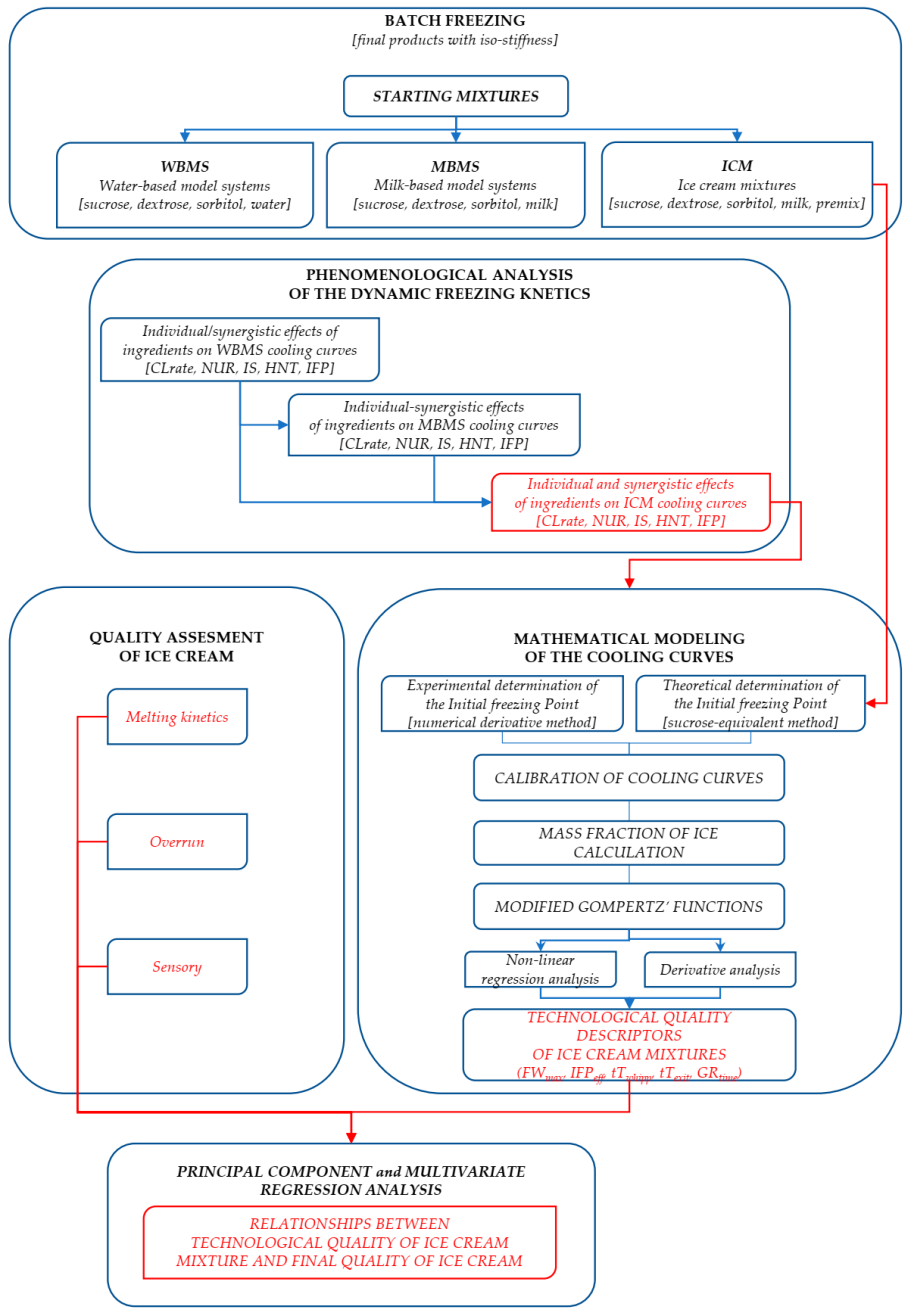
Flow diagram that explains the experimental methodology.

The experimental strategy is reported in general terms in the (Scheme 1) and includes the following steps:

- preparation of mixtures characterized by different total solid contents and different level of complexity (water-based model systems, milk-based model systems and real ice cream mixtures);
- partial freezing of the starting mixtures inside a batch SSHE;
- phenomenological analysis of the behavior of model systems and ice cream mixtures in order to explore the effects on the solute-solvent interactions on the main freezing events registered along the experimental cooling curve (it was necessary to simplify the mathematical description of the water crystallization kinetics occurring under the SSHE conditions);
- numerical analysis of the cooling curves (temperature calibration and mathematical modeling of the water crystallization kinetics;
- statistical analysis to select the most significant kinetic properties able to classify the ice creams according to quality characteristics such as overrun and melting and sensory properties;
- development and validation of statistical models able to predict the melting and sensory attributes of the final ice cream.

Two sugars (sucrose and dextrose) and a polyol (sorbitol) of interest as sweeteners in the ice cream production were selected for their different colligative properties to modulate both the equilibrium temperature and the crystallization kinetic. The individual and synergistic colligative effects of such sugars were firstly evaluated in the water- and milk-based solutions. Then, the information acquired with these trials were used to clarify the dynamic behavior of the investigated ice cream mixtures. 

### 2.1. Sugar-Based Model Systems

Three water-based model systems, namely “WSUCR,” “WDEXT”; and “WSORB” were produced by adding 300 g of sucrose or dextrose or sorbitol to 1000 g of distilled water. 

Similarly, three milk-based model solutions were prepared by replacing water with whole milk. In the following they are referred as to “MSUCR,” “MDEXT,” “MSORB,” respectively.

Another more complex water-based model system, namely “WSUCR + WSORB, was produced by adding 150 g of sucrose and 150 g of sorbitol to 1000 g of distilled. 

Similarly, a more complex milk-based model system, namely “MSUCR + MDEXTR,” was produced by adding 150 g of sucrose and 150 g of dextrose to 1000 g of whole milk. 

All ingredients were purchased from REIRE s.r.l. (Reggio Emilia, Italy). About 10 Kg of each of the 5 solutions were prepared according to the following procedure. The ingredients were blended under mechanical agitation carried out with a turbomixer (Robot Coupe-94305 Vincennes Cedex-France) for 1 min, then the solution was heated up to 88 °C (the temperature was reached in about 5 min) and finally cooled under 8 °C (the temperature was reached in about 1.5 min).

Table 1 describes the precise formulation of the model systems. 

In order to exactly quantify the initial water content and the colligative and not-colligative compounds derives from the ingredients used, the mass balances of all the model systems were calculated and reported in (Table 2). 

As can be inferred from these data, the water concentrations were in the ranges 77.03–78.31 and 69.53–70.81 g/100 g for water-based and milk-based model systems, respectively while sugars were in the range 21.69–22.97 and 25.35–26.62 g/100 g for water-based and milk-based model systems, respectively. In the milk-based systems, the concentrations of fats, milk solids non-fat (MSNF, which includes proteins and ashes from whole milk, skim milk powder and milk proteins) and NaCl were 1.19, 2.56 and 0.10 (g/100 g), respectively. 

### 2.2. Ice Cream Mixtures

All the ice cream mixtures were prepared with variable amounts of sucrose, dextrose, sorbitol but with the same amount of whole milk and of a multicomponent basis. The composition of the multicomponent basis was the following: skimmed-milk powder (30%), dextrose (30%), maltodextrin (18.36%), inulin (10%), milk proteins (5%), sucrose esters (4.35%), carrageenan (0.29%), Tara-seed flour (1.5%) and carboxymethylcellulose (0.5%). All ingredients were purchased from REIRE s.r.l. (Reggio Emilia, Italy). 

First of all, the following 3 ice cream mixtures were prepared with the increasing concentrations of the total sugar content (expressed as g/100 g of water): 16.6% (namely LSC, low sugar content); 23.1% (namely MSC, medium sugar content), whose composition corresponds to that of the traditional recipe for the Italian-style ice cream; and 28.6% (namely HSC, high sugar content). Further 4 MSC ice cream mixtures, namely MSC1, MSC,” MSC3 and MSC4, were prepared by varying the relative amounts of each sugar without changing the total sugar content (Table 3). 

The mass balances of all the ice cream mixtures were reported in (Table 4). 

The concentrations of water were 76.01 and 65.42 (g/100 g) for LSC and HSC, respectively and ranged from 70.20 to 70.41 within all the MSC mixtures. Sugar concentrations were 18.34 and 29.74 (g/100 g) for LSC and HSC, respectively and were within 24.37–24.58 (g/100 g) range for all MSCs. The concentrations of fats were 1.32 and 1.13 (g/100 g) for LSC and HSC, respectively and 1.22 (g/100 g) for all MSCs. MSNF were 4.20, 3.60 and 3.88 (g/100 g) for LSC, HSC and MSCs, respectively. Finally, NaCl contents were 0.14 and 0.12 and 0.13 (g/100 g) for LSC, HSC all MSCs, respectively.

For each of the ice cream mixtures denominated LSC, MSC and HSC, an amount of about 60 Kg was prepared. Instead, for each of the ice cream mixtures denominated MSC1, MSC2, MSC3 and MSC4, an amount of about 30 Kg was prepared. The ingredients were blended under mechanical agitation carried out with a turbomixer (Robot Coupe, Vincennes, CEDEX-France) for 1 min, then the solution was heated up to 88 °C (the temperature was reached in about 5 min) and finally cooled under 8 °C (the temperature was reached in about 1.5 min). 

### 2.3. Aging Operation

Model systems and ice cream mixtures were held at 4.0 ± 0.2 °C for 24 h under static conditions in a cabinet (Multispace 800, Electrolux, Porcia-Pordenone, Italy). Such operation allowed the partial crystallization of the milk fat globules and the protein hydration. Both physical phenomena concur to the formation of a viscoelastic network inside the unfrozen phase during freezing. The aged products were then homogenized with a turbomixer (Robot Coupe-94305, Vincennes, Cedex-France) for 1 min before freezing.

### 2.4. Freezing and Tempering Operation 

**Scheme 2:**
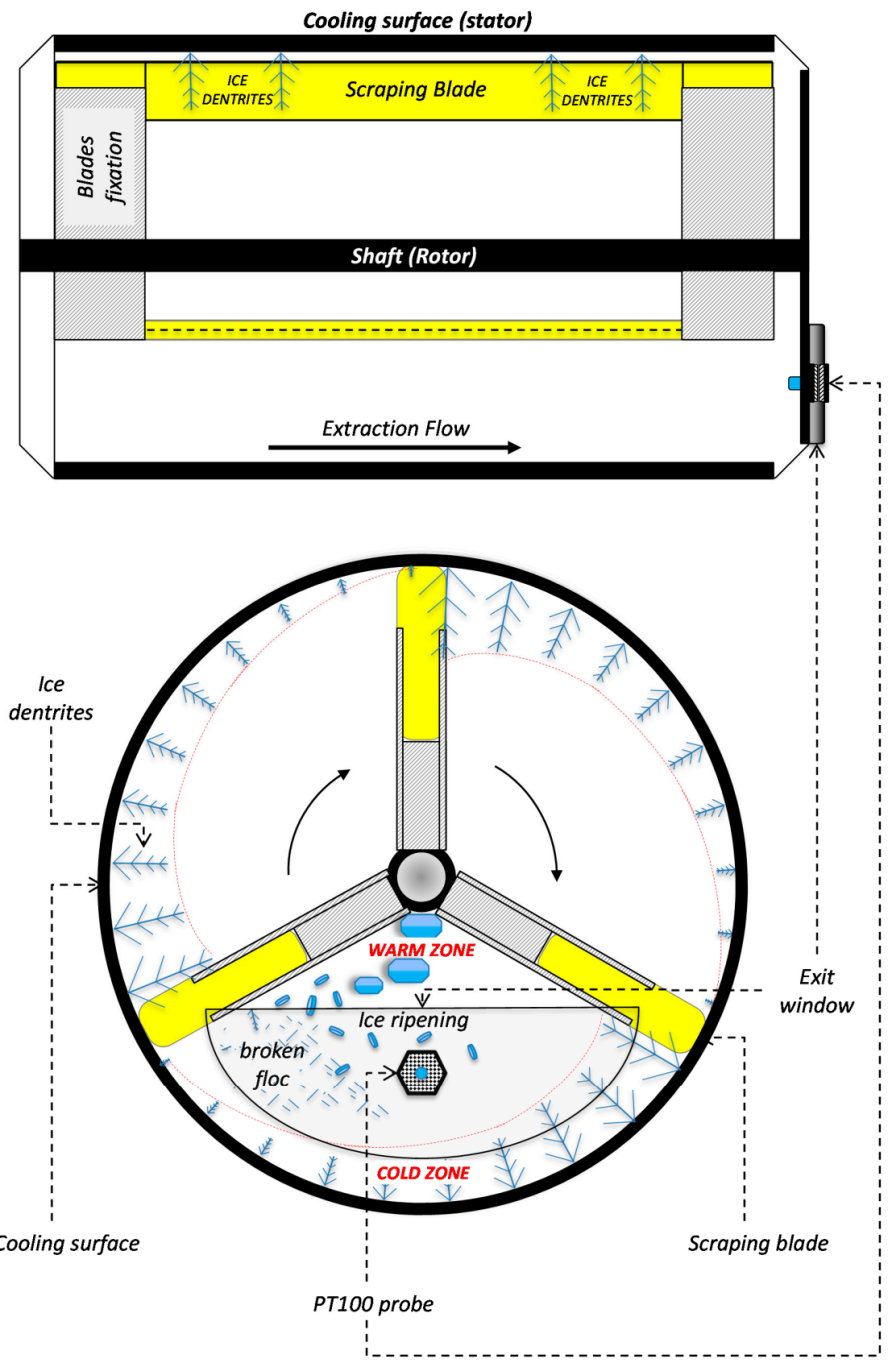
Horizontal Surface-Scraped Heat Exchanger (SSHE) used for batch ice cream production.

The freezing operation was carried out on batches of about 3 Kg of aged model systems or ice cream mixtures in a SSHE (model Compacta 3003 RTX, Carpigiani, Bologna, Italy) having the following technical characteristics: capacity 7.5 Kg; max power 7.8 kW; 3 blades rotating at 150 rpm; and 1.1 Kg of R404A as coolant. The temperatures were recorded by using a PT100 probe (TRC, Italcoppie Sensori, Malagnino, Italy) with a DIN sensor certified according to the international standard IEC751 for its insensibility to the electrical interferences, response time below 3.5 s (i.e., the time taken for the sensor to react to 50% of step change in temperature), platinum resistance of 100 Ω 0 °C and accuracy of ±0.03 °C at 0 °C. The probe worked at an operative frequency of 1 Hz. The probe was located inside the SSHE at an intermediate radial distance between the axial shaft and surface wall, within the gap between the scraping blades and freezer exit according to the scheme reported in (Scheme 2). 

The probe location was chosen in order to obtain comprehensive information about the ice crystallization kinetics, taking into account that the ice nucleation rate is higher on the surface of the SSHE (which is the colder zone) and that the ice growth and air whipping rates is higher inside the SSHE bulk (which is the warmer zone) [12].

**Figure 1:**
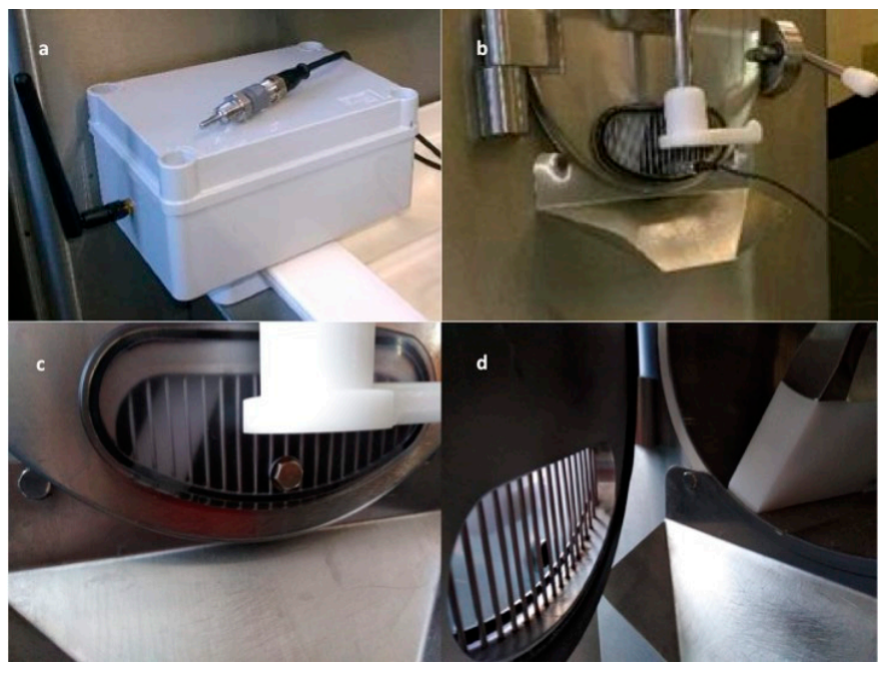
Installation of the temperature probe: PT100 and wireless device (**a**); external (**b**,**c**) and internal (**d**) view of the process connection on the exit window of the SSHE.

The temperature probe was equipped with a steel-sealing standard clamp (G1/2 units), a fixing element qualified for process connection. It was fixed on the Plexiglas exit window of the SSHE through a horizontal installation (Figure 1). The position of the probe was hold during the entire cooling process before opening of the extraction window at the end of process, allowing the ice cream to exit from the SSHE. Time—temperature data were transmitted to an external computer by means of a 24-bit ADC wireless device.

In order to minimize the variability of the freezing experiments, after homogenization, the aged liquid products were immediately transferred to the SSHE dasher, whose temperature was previously regulated at 12 °C.

During freezing the mixture mechanical stiffness increased due to temperature lowering, ice crystallization and unfrozen phase concentration. The final product was extracted from the SSHE after it reached a mechanical stiffness corresponding to the 65% of the maximum motor electric power.

Tempering operation was necessary to stabilize the ice cream texture before melting and sensory analysis. To this aim, immediately after extraction from the SSHE, ice creams were held at −13 ± 0.2 °C for 24 h in a ventilated ice-cream cabinet (Millennium, Isa Bastia Umbra, Perugia, Italy).

### 2.5. Calculation of the Theoretical Freezing Point

The theoretical freezing point is usually defined as the sub-zero temperature at which the water begins to crystallize in a solution of colligative solutes. Freezing point depression of both model systems and ice cream mixtures was estimated considering their contents in colligative solutes, according to the well-established sucrose equivalent method [5]. In order to consider the contribution of the different sugars contained in the formulations, each of them was converted in sucrose equivalents, that is, the equivalent concentrations of sucrose that would have the same freezing point depression effects in a pure sucrose solution. With this aim, the mean molecular weight of all each individual colligative solute was divided to that of sucrose, then the result was multiplied for the mass fraction of the colligative solutes as expressed in g/100 g of water in the investigated solution. The contributions of all colligative sugars, MSNF and NaCl were considered. More specifically, the colligative sugars derive from both the added sweeteners, that is, sucrose, dextrose and sorbitol and from the other ingredients that include the milk and/or the multicomponent basis. Table 5 and Table 6 list the sucrose equivalents of model systems and ice cream mixtures, respectively. 

Then, the contribution of sugars to the freezing point depression was calculated using the polynomial equation with intercept through zero derived from the regression model where g sucrose/100 g water is graphed against freezing point depression [5]. 

The contributions of MSNF and NaCl to the freezing point depression were calculated according to Goff and Hartel [5] and Potter et al. [19], respectively. 

Fats, proteins, large molecular weight carbohydrates (except inulin and maltodextrins), stabilizers and emulsifiers do not contribute to freezing point depression since fats are immiscible with the aqueous phase and proteins and polysaccharides are very large molecules [5] with a negligible capacity to bind water molecules with respect to monosaccharides, disaccharides, sugar alcohols and salts. 

### 2.6. Ice Cream Analysis

#### 2.6.1. Overrun

In order to evaluate the air volume incorporated within the final product, the following formula was applied:

(1) $$\mathrm{OVERRUN}=\;100\cdot \left[\frac{W_{\mathrm{mixture}}{\;-\;W}_{\mathrm{ice}\;\mathrm{cream}}}{W_{\mathrm{Ice}\;\mathrm{cream}}}\right].$$

A calibrated metal cup of 235 mL ± 0.1 mL was used for the measurements.

#### 2.6.2. Melting Test

Melting tests were carried out in a conditioned chamber at 20 ± 0.1 °C using a known volume of ice cream contained in a calibrated cup. The ice cream sample was transferred on a 2 mm mesh sieve under which there was an empty cup, placed on a precision balance (Gibertini Europe 500, Milano, Italy), to collect and weigh the ice cream that melts and drops under gravity. The weight of the melted ice cream was registered at the first drop and after 15, 30 and 45 min. 

#### 2.6.3. Sensory Evaluation of Ice Cream 

A trained panel of 12 judges was selected to perform a rating analysis of the ice cream based on their perception of differences among samples and rating for intensity of the sensory attributes according to attribute-specific hedonic scales as described in (Table 7):

A pre-screening questionnaire was administered for recruitment purposes to 35 undergraduates of the Department of Life Sciences, University of Modena and Reggio Emilia (Italy) who had followed a 10 h course on ice cream sensory evaluation. Interest and availability, lactose-tolerance, natural dentition, normal health conditions, abstinence from smoking, alcohol and chewing betel and no dietary restriction on calorie intake were included in the pre-screening questionnaire as major criteria for recruitment. Thirty people compliant with the pre-screening criteria were then recruited as aspiring panelists and attended a one-year training program to improve their capability to identify and rank the intensity of sensory attributes of the ice cream in training tests sessions of 5 days every month. A set of ice cream references were prepared with four levels intensity for each sensory attribute of interest and used for training purposes. Such reference samples were prepared using the same ingredients and freezing conditions used to produce the MSC ice cream if not otherwise specified. However, the total amount of the added sucrose or emulsifiers and stabilizers present in the multicomponent basis was modulated according to a linear increasing function. Concerning sandiness attribute, the reference samples were produced with the same MSC composition but tempering operation was performed under fluctuating temperatures conditions around the tempering target (−13 °C) by shutting off the power supply intermittently for increasing time periods on 24 h. For cool sensation, reference samples were produced with the same MSC composition but were extracted from the SSHE at four drawing temperatures. To evaluate both flavor persistency and intensity as well as off-flavor, reference samples were produced with the same amount of total added sugars of MSC ice cream but with different ratio between whole milk and multicomponent basis.

The specifications used to produce the reference ice creams and their intensity levels used for the rating tests are listed in (Table 8). 

During training sessions, the reference ice creams were presented in a random order and the judges were asked to pick the samples with different intensity and rank them according to the perceived intensity using the attribute-specific hedonic scale. To compensate for the known variations throughout training test sessions a qualifying test was performed, those who make an acceptable (75 percent correct) were chosen, otherwise the training session was replicated. A series of 12 judgments were obtained from each prospective panelist. Twelve judges were selected as panel components based on their abilities to match at least 5 out 8 of sensory attributes and at least 3 out 4 of rating tests.

During both the training and rating tests sessions, the ice creams were removed at −13 ± 0.2 °C from the ventilated cabinet used for tempering and anonym glasses of about 100 mL were prepared containing the same amount of product. No more five ice cream samples were analyzed for each test session. Panelists were asked to firstly evaluate the appearance of the ice cream surface, noting if the ice creams have retained its form and shape. Next, they assessed the in-mouth properties. Immediately after placing a portion of ice cream into the mouth, they roll it between the incisors and bring them together very gently, noting the eventual presence of ice crystals and whether grittiness is apparent between the teeth. A small portion between the incisors revealed the presence of minute traces of a gritty or sandy texture. Meltdown intensity was assessed by evaluating the time required for the product to soften and melt in the mouth when continuously pressed by the tongue against the palate. When the sample was completely liquefied and warmed to body temperature, the detection of the sweetness as well as of flavor intensity or persistency was done. It was accomplished by placing a small teaspoon of the ice cream directly into the mouth, quickly manipulating the sample between the teeth and palate and noting the taste and odor sensations as well as any releasing of after taste. 

Since the evaluations of each attribute were carried out on evaluation scale specific to that attribute, the scores assigned by the panelists were re-parameterized on a common scale by performing standardization of the original scores to show the overall ice-cream profile in a single figure for comparative purposes. To eliminate both the units and scaling effects each score was divided by the standard deviation, obtaining a variance of 1 but different means and ranges. 

To evaluate the preference among the investigated ice creams, both the highest scores and optimum score (where defined) were fixed as the highest level of liking degree. The final judgment was calculated by summing all desired sensory attributes (i.e., appearance, cool sensation, sweetness, meltdown, flavor intensity and flavor persistency) and subtracting all undesired ones (i.e., sandiness and off-flavor). As a consequence, an overall liking level was calculated:

(2) $$\bar{\mathrm{LIKING}\;\mathrm{LEVEL}}\;=\;\sum_{J=1}^{12}\left\{100\cdot \frac{\sum {\mathrm{score}}_{j}^{i}-{\mathrm{score}}_{j}^{d}}{\sum {\mathrm{score}}_{\max}^{i}}\right\}/12$$

where ${\mathrm{score}}_{j}^{i}$ was the experimental score provided by the jth judge for the ith attribute; ${\mathrm{score}}_{\max}^{i}$ was the maximum score for each ith attribute and ${\mathrm{score}}_{j}^{d}$ was the experimental score assigned by the jth judge to the dth off-flavor attribute.

#### 2.6.4. Data Preprocessing and Statistical Analysis

Model systems were produced in triplicate. Concerning the ice cream mixtures, 18 replicates were produced for the samples denominated LSC, MSC and HSC and 9 replicates were produced for MSC1, MSC2, MSC3 and MSC4 samples. 

To reduce the experimental noise, time-temperature data were filtered by using the 3-point Savitzky-Golay numerical method [20].

The reproducibility of the time-temperature data was evaluated by overlapping the cooling profile of the independent replicates. More than 1200 values (400 sampling times for each temperature profile) were used for the descriptive statistical analysis of the differences measured among three replicates. As calculated by doubling the experimental standard error, the uncertainty of temperature measurement always ranged within the +/−0.2 °C.

Principal component analysis (PCA) was performed to classify the ice cream samples according to composition and freezing properties. Multiple regression analysis (MRA) was carried out to develop predictive models for the following ice cream quality properties: melting rate, overrun, appearance, cool sensation, smoothness, sweetness, meltdown, flavor intensity and flavor persistence. The predictive accuracy of the MRA models was evaluated by performing the cross-validation on an independent dataset. The original dataset was split in two subsets namely “calibration dataset” (including 70% of the samples of the original dataset) from which the MRA models were derived and “validation dataset” (including 30% of the samples of the original dataset) against which the MAR models were validated. The goodness of fit for MRA models and for the linear regression between predicted vs. observed data was evaluated calculating the multiple R^2^ with a statistical significance of *p* < 0.01.

All statistical analyses were carried out using Statistica 10.0 (StatSoft 2011; Tulsa, OK, USA).

## 3. Results and Discussion

In this section, the cooling curves registered during the freezing of the model solutions and of the ice cream mixtures were separately analyzed and discussed.

### 3.1. Cooling Curves for Water-Based Model Systems

**Figure 2:**
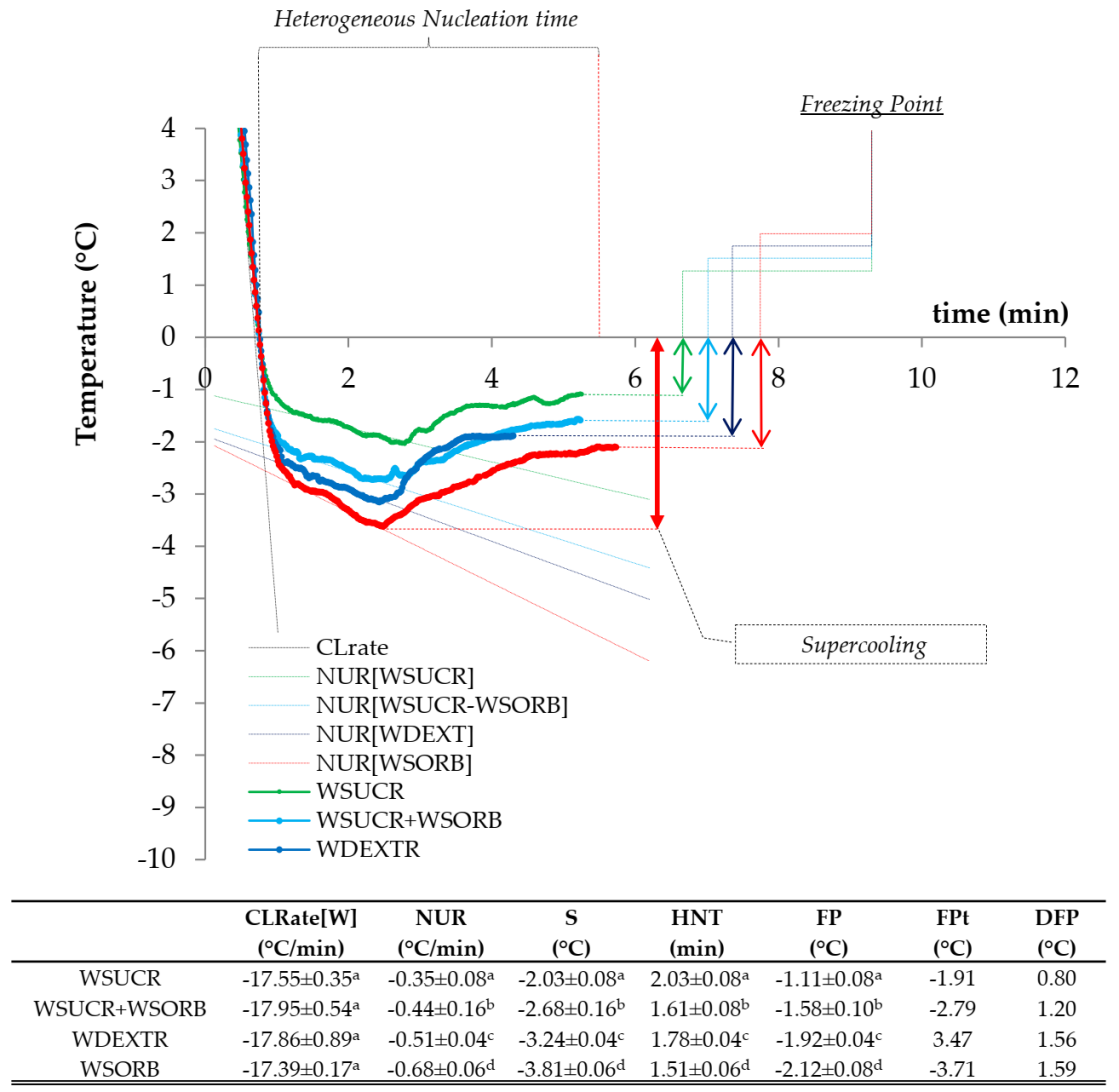
Cooling curves of the water-based model systems. The absolute uncertainty of 0.18 °C was considered for FPt according to Jaskulka et al. [24]. In columns, different letters indicate significant differences (*p* < 0.05) among the water-based model systems.

Figure 2 shows the cooling curves of the 4 water-based model solutions together with their thermodynamic and kinetic properties.

As can be inferred from figure, a similar trend can be recognized among the model systems with a significant difference. Temperature decreased under 0 °C with a chilling rate (CL_rate_) of −17.69 ± 0.52 °C/min. The lowest temperature in the cooling curve represents the supercooling (in the figure it was referred to as “S”): it was reached by all the investigated water-based model solutions on average after about 2.4–2.7 min. 

Since the supercooling event was unique along with the cooling curve, the nucleation rate (NUR) was determined as the slope of the linear regression analysis of the cooling curve in the time domain ranging between TP1 and S. The formation of unstable clusters of ice nuclei started at about 0.8 min and NUR was on average −0.49 °C/min.

Starting from supercooling, the formation of new metastable nuclei occurred with phase transition with the release of the latent heat that determines the asymptotical increase of solution temperature up to the equilibrium level, representing the freezing point (in the Figure 2 it was referred as to FP). The hydrogen bond formation required to entrap free water molecules to form clusters (ice embryos) is an exothermal process without phase transition that determines a significant increase in the cooling temperature rate. The experimental freezing point represents the equilibrium temperature required by the water-based solutions to form stable clusters of ice crystals able to growth under SSHE dynamic conditions. From this point, the water crystallization follows a steady-state kinetics (i.e., without changes in temperature). 

The “Heterogeneous Nucleation time, HNT” was calculated as the time length between TP1 and FP. HNT values were in the range 1.51 ± 0.06–2.03 ± 0.08 min. 

The kinetics (NUR, S) and thermodynamic (FP) parameters were sugar-specific and followed the order WSUCR, WSUCR + WSORB, WDEXTR and WSORB. This behavior could be attributed to the different ability of disaccharides, monosaccharides and sugar alcohols to form hydrogen bonds with water molecules (colligative behavior), which is in turn related to the number of hydroxylic groups and the sugar steric hindrance. These results are in agreement with the findings of previous research. The nucleation rate in dextrose solutions shows a low-order dependence upon supercooling, for supercooling in the range −0.25 °C and −1.00 °C but it increases markedly with increasing sugar concentration [21]. Sucrose significantly slows down the nucleation rate [22]. The highest hygroscopicity of sorbitol resulted in the lowest FP with respect to other reducing sugars [23]. 

Sugars were the only colligative solutes determining the depression of the freezing point in the water-based model systems. The theoretical freezing point FPt for the water-based model systems was calculated according to the sucrose equivalent method [5] using the freezing point depression values tabulated for pure sucrose solutions and the “sucrose equivalency” between the compositions was derived from a comparison of the mean molecular weight for the colligative solutes present (in this case only sucrose, dextrose and sorbitol), with that of pure sucrose (Table 9). 

As can be inferred from the data reported in (Figure 2), FPt values were significantly lower than the experimental (FP) ones. Furthermore, differences (∆FP) between the FPt and FP increased according to the sugar ability to form hydrogen bonds, that is, sucrose < dextrose < sorbitol. ∆FP were mainly attributed to the temperature probe location, that is, at the intermediate position between the colder (freezing surface) and the warmer zone (rotor shaft) of the SSHE. Based on experimental evidences, Goff and Hartel [5] concluded that the temperature profile inside a continuous SSHE freezer for ice cream production is not uniform, for example, radially, the temperature increases on average from −26 °C at the wall to up to −28 °C in the bulk fluid at the center. During their rotation, the scraping blades enables the mixing between the clustered nuclei arising from the cold wall and the unfrozen serum phase in the warmer bulk zone, thus a time-dependent temperature gradient takes place between the freezing surface and rotor shaft. The rate at which the temperature gradient is established depends on the rotational speed and on thermal diffusivity and viscosity of the freezing mixture. No literature data are available about horizontal batch SSHE used in ice cream productions. In the present study, the rotational speed of the scraping blades was 150 rpm and the time-to-response of the PT100 probe was 3.5 s. This means that after 3.5 s, the rotor shaft accomplished for about 9 complete rotations, enabling fast mixing and shearing of the ice cream mixture but without complete loss in temperature gradient. At the location chosen, the PT100 measured an apparent equilibrium temperature higher than that occurring on the freezing surface, which would be close to the theoretical initial freezing point. A proximate descriptor of the temperature gradient in the batch SSHE can be obtained by doubling the ∆FP value, with results very close to those documented for continuous SSHE. However, additive contributions to ∆FP were also considered, for example, SSHE’s dynamic conditions and non-ideal behavior of the investigated sugar-rich solutions. Scraped blades rotating at high-speeds strongly interfere with the water crystallization: it resulted in an enhancement of the heat transfer coefficient and therefore in the decrease of supercooling required for the nucleation, so that ice crystallization occurred at higher temperatures than in the static freezing conditions considered to calculate the theoretical freezing point. Similar results have been obtained in studies focused on the ice crystallization in brine solution inside a batch SSHE. The values of ∆FP were 0.80, 1.20, 1.56 and 1.59 °C for WSUCR, WSUCR + WSORB, WDEXTR and WSORB, respectively. to Higher sugar-water interactions determined higher liquid viscosity. In these conditions, the lower shear rate inside the liquid bulk determined lower rates of mixing and heat transfer from the coldest point (freezing surface of the SSHE) to the location of the temperature probe.

### 3.2. Cooling Curves for Milk-Based Model Systems

**Figure 3:**
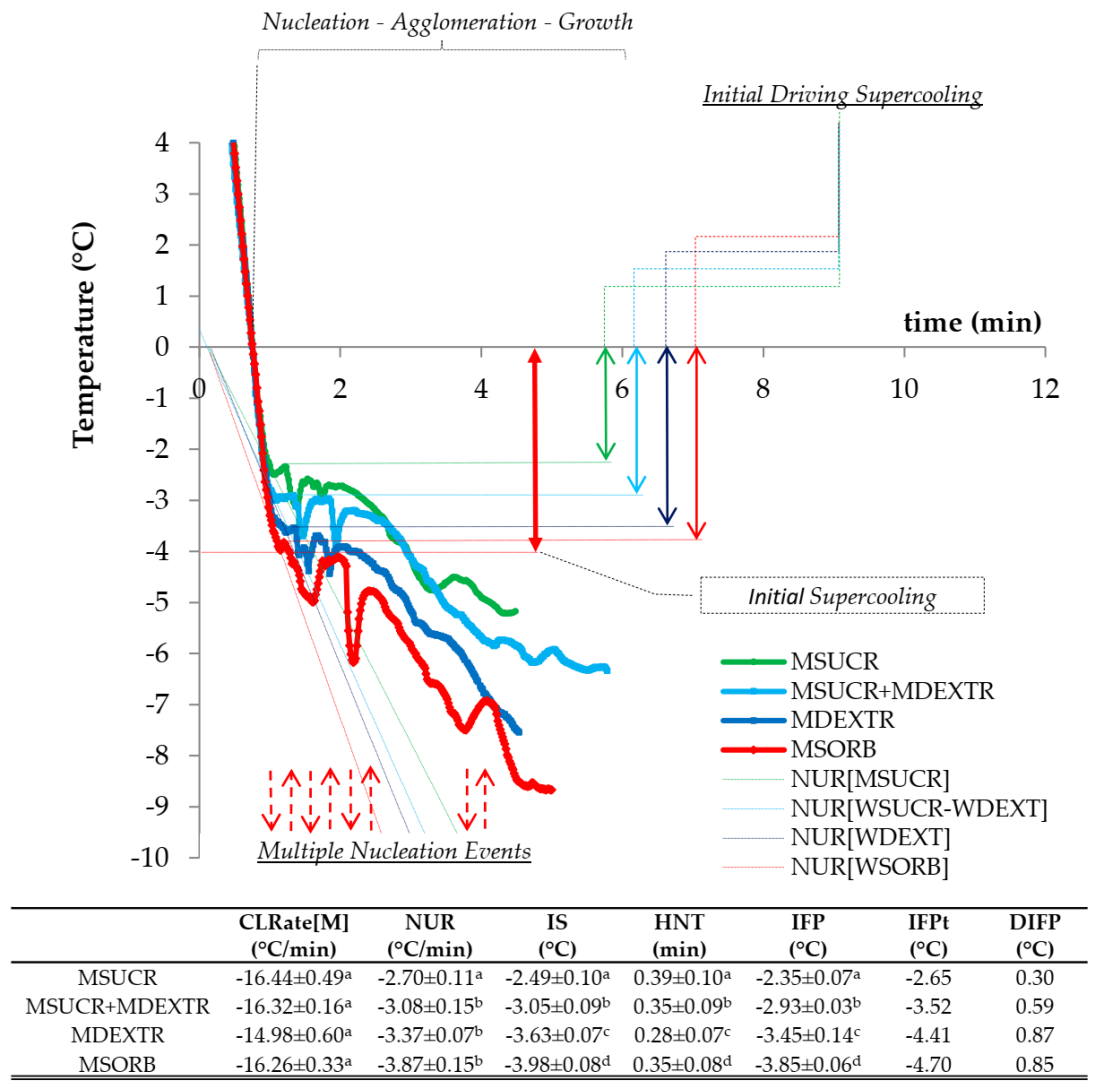
Cooling curves of milk-based model systems. The absolute uncertainty of 0.18 °C was considered for IFPt according to Jaskulka et al. [24]. In columns, different letters indicate significant differences (*p* < 0.05) among the milk-based systems.

Figure 3 reports the cooling curves of the four milk-based model solutions together with their thermodynamic and kinetic properties. 

As can be inferred from data, alternate exothermic and endothermic events characterized the cooling process suggesting that more than one apparent nucleation steps occurred with an equal number of supercooling events. Dynamic and synergistic interactions occurring at a molecular scale between water and sugar molecules and at a colloidal scale between ice crystals and between sugars and milk constituents were hypothesized as the main causes of the observed temperature profiles and they could be due to the formation of hydrogen bonds. The formation of large agglomerates of ice crystals was observed in freezing experiments concerning sugar solutions [25]. 

As can be inferred from figure, the temperature decreased under 0 °C with a CL_rate_ of −16.00 ± 0.52 °C/min and was significantly lower than that observed in water-based model systems. The presence of milk fat and proteins is responsible for a relatively higher liquid viscosity and a lower heat transfer coefficient of the milk-based model systems. Due to the occurrence of several apparent nucleation events, the temperature corresponding to the first event was determined as initial supercooling, IS. Starting from the initial supercooling, the formation of new metastable nuclei occurred during phase transition with the release of latent heat that determines an increase of solution temperature up to the first equilibrium level, that is, the experimental initial freezing point, IFP. The theoretical freezing point, IFPt, for the milk-based model systems were significantly lower than the experimental ones (Figure 3). However, the difference between IFPt and IFP values, ∆IFP, was on average significantly lower than that observed in the water-based model systems: ∆IFP were 0.30, 0.59, 0.87 and 0.85 °C for MSUCR, MSUCR + MDEXTR, MDEXTR and MSORB, respectively. An additive contribute of the colloidal milk proteins and salts to the heterogeneous nucleation process was hypothesized, resulting in a decrease of supercooling required for the nucleation, so that the water crystallization occurred at higher temperatures than in the static freezing conditions considered to calculate the theoretical freezing point. Experimental evidences were provided by the fact that, differently to the water-based model systems, the experimental temperatures corresponding to the IS were always higher than the corresponding IFPt (the theoretical freezing point is calculated accounting for sucrose equivalency without ant protein contribute to the equilibrium temperature). 

Due to the occurrence of more than one supercooling events, the nucleation rate (NUR) was determined as the slope of the linear regression analysis of the cooling curve in the time domain ranging between TP1 and IS; while the “Heterogeneous Nucleation time, HNT” was calculated as the time length between TP1 and IFP. NUR was on average −3.25 °C/min and the HNT values were in the range 0.28 ± 0.07–0.39 ± 0.10 °C/min indicating that the IFP point was reached faster than in water-based models. 

Both the kinetic (IS) and thermodynamic (IFP) parameters highlighted sugar-specific effects on the water crystallization kinetics and were in the following decreasing order MSUCR, MSUCR + DEXTR, MDEXTR and MSORB. 

### 3.3. Cooling Curve for the Ice Cream Mixtures

As for the milk-based systems, more than one apparent nucleation steps occurred with an equal number of supercooling events but they occurred in a defined time and with lower temperature fluctuations due to the presence of emulsifiers, which promoted the destabilization of the milk fat globules and stabilizers, responsible for the higher initial viscosity. After heterogeneous nucleation, that is, an unstable crystallization period during which temperature fluctuation may be registered due to more than ones supercooling and nucleation, the ice nuclei reach a minimum stable size, enough to growth at a macroscopic scale size without changes in temperature. Such equilibrium temperature was kept for very little time, because the significant subtraction of water as ice from the liquid mixture caused the concentration of the unfrozen phase that becomes slurry. Water crystallization required lower temperatures to progress when the unfrozen phase concentrates significantly.

In order to simplify the quantitative analysis of the water crystallization kinetics, the following main steps of the cooling curve were individuated:

Chilling, corresponding to the linear part of the cooling curve above 0 °C.

Initial Freezing Point (IFP) corresponding to the highest temperature (i.e., the first equilibrium temperature) reached after the first supercooling (kinetic event)

Steady-State, part of the cooling curve where the equilibrium temperature remains constant for a little time since the ice crystallization occurs without significant increase of the concentration of the unfrozen phase of the ice cream mixture.

Effective Freezing Point (IFP_eff_) corresponding to the equilibrium temperature, at which the water crystallization kinetics is at its acceleration point and the ice starts to accumulate significantly and extensively in the ice-cream mixtures. 

Dynamic Nucleation, part of the cooling curve below 0 °C and IFP_eff_ corresponding to the multiple heterogeneous nucleation (supercooling) events.

Dynamic Ice Crystallization, part of the cooling curve between IFP_eff_ and temperature at which the ice cream exits (T_exit_) from the SSHE where the ice crystals growth is limited by the length of the freezing time as a balance between the mechanical shearing and technological properties of the ice cream mixture 

**Figure 4:**
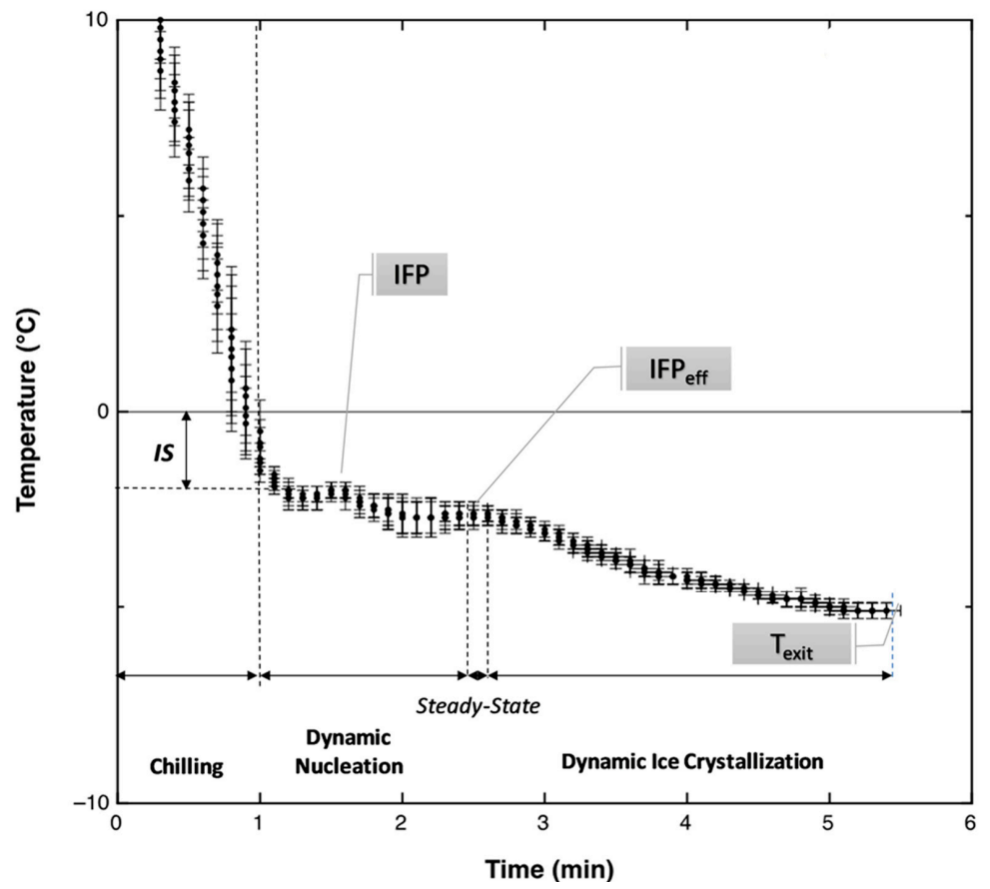
Cooling curves of the HSC ice cream mixtures.

As an example, (Figure 4) reports the cooling curves of the HSC ice cream mixtures. 

Since more than one nucleation events were observed in the ice cream mixtures during cooling, both the NUR and HNT were calculated as for the milk-based model systems. The kinetic and thermodynamic parameters of the HSC ice cream mixtures were compared to those of the milk-based model systems since they were prepared with the same amounts of added sugars. CL_rate_ (−15.41 ± 1.60 °C/min) was not significantly different from those referred to the milk-based model systems, while NUR (−7.80 ± 1.42 °C/min) and HNT (0.79 ± 0.16 min) were significantly higher. Regarding IS (−2.34 ± 0.18 °C) and IFP (−2.23 ± 0.35 °C), no differences were highlighted between HSC and MSUCR, while the values found for the ice cream were significantly lower than those observed for MSUCR + MDEXTR, MDEXTR and MSORB. The temperature gradient (0.70 ± 0.17 °C), expressed in terms of the ∆IFP parameter was not significantly different to that (0.65 ± 0.27 °C) measured on in milk-based model system. However, HSC samples showed the highest average value (1.39 ± 0.17 °C) of ∆IFP: it was not significantly different from that (1.29 ± 0.37 °C) measured in the water-based model systems. The behavior observed for the HSC samples was attributed to the highest concentration of the hydrocolloids of the multicomponent basis used in their formulations. The stable incorporation of finely disperse air cells surrounded by milk proteins and partially-destabilized fat globules concurred to the heterogeneous nucleation process, resulting in a decreasing of the supercooling required, so that water crystallization occurred at higher equilibrium temperatures than in the static freezing conditions considered to calculate the theoretical freezing point.

The theoretical values of IFPt for the ice cream mixtures were estimated considering the presence of sugars, MSNF and NaCl, whose individual contributions are listed in (Table 10).

### 3.4. Solute-Solvent Interactions

**Figure 5:**
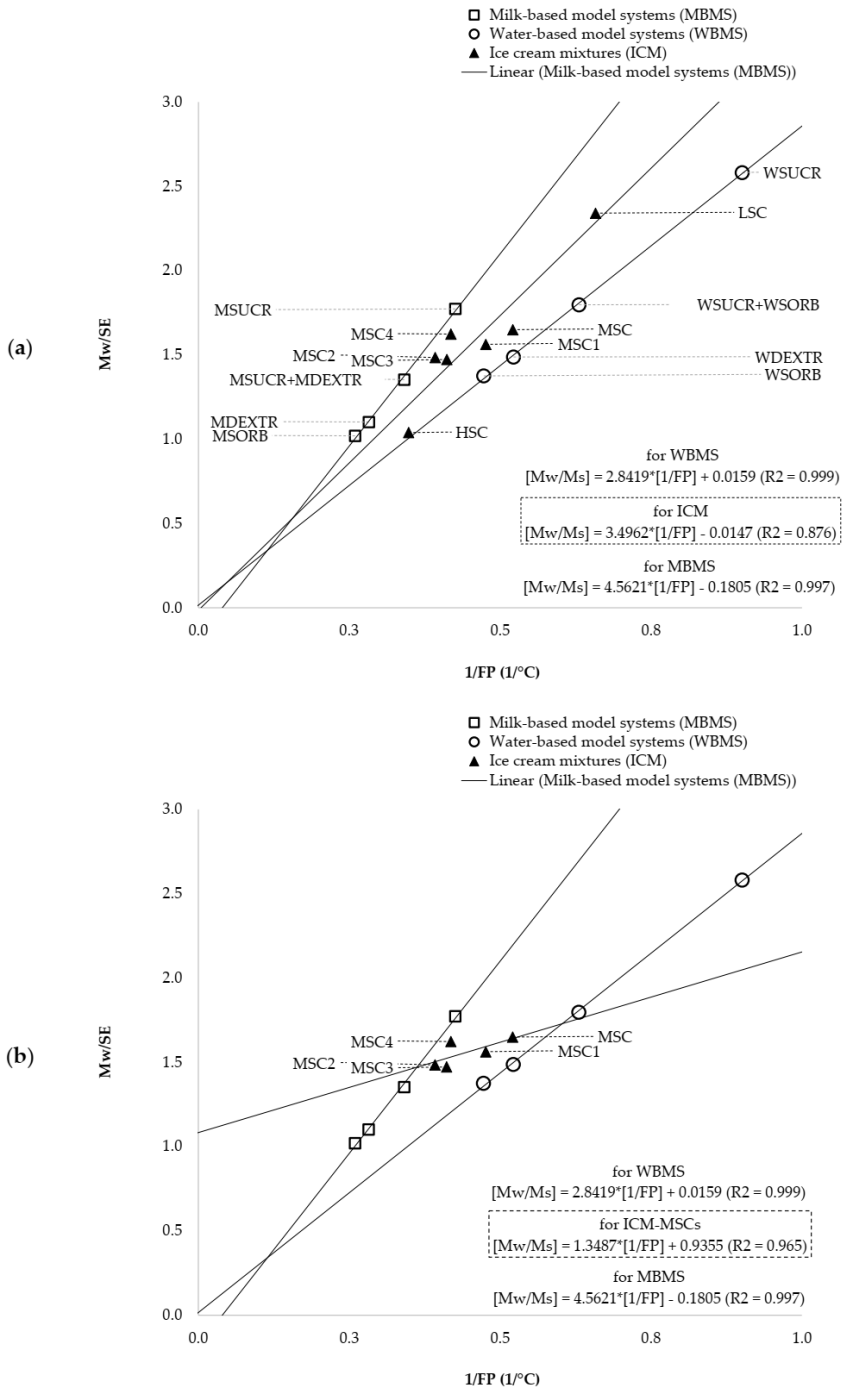
(**a**) Interaction Linear Plot for water-based, milk-based models and ice cream mixtures. The major contribute of the hydrocolloids on the colloidal scale is easy to detect (**b**) by comparing the model systems against the ice cream mixtures (ICM-MSC) having the same total solids and with lower sugar content.

Figure 5 shows the results deriving from the Interaction Linear Plot (ILP) analysis of model systems and ice cream mixtures that was performed using Weast’s equation for non-ideal solutions [23]. The *x*-axis accounts for the inverse absolute values of the experimental freezing point (it is referred as to 1/FP, (1/°C)). The lower values of the *x*-axis correspond to the higher freezing point depression. The *y*-axis accounts for the ratio Mw/Ms (where Mw and Ms are respectively the initial concentration of water (expressed as g/100 g of the starting solution) and the concentration of sucrose equivalents (expressed as g/100 g of water) corresponding to the colligative solutes sugars, MSNF and NaCl. The strength of colligative solute-solvent interaction is represented by the intercept of the linear regression plot while the degree of dependence between the freezing point and Mw/Ms is represented by the slope. As can be inferred from the figure, the linear Weast’s equation is satisfactorily applied to all the investigated mixtures: the inverse value of the freezing point increased linearly with Mw/Ms (with *R*^2^ > 0.876 at least). 

The absolute values of both the slope and intercept from ILPs were 2.8419 and 0.0159, 3.4962 and 0.0147, 4.5621 and 0.1805 for water-based model systems, ice cream mixtures and milk-based model systems, respectively. Intercept data suggested that the greatest contribution to the overall strength of the interaction between the colligative solutes and the free water molecules was observed in milk-based model systems, more likely caused by the milk proteins and partially destabilized fat globules at a colloidal scale. From one hand, both milk proteins and partially destabilized fat globules behave as hydrophilic colloids: they can subtract free water molecules, resulting in an increasing of the concentration of sucrose equivalents in the unfrozen serum phase and therefore in a decreasing of the initial freezing point. Furthermore, an additive sugar-specific colligative effect arising from lactose, MSNF and milk salts concurs to the degree of dependence of Mw/Ms on freezing point depression. On the other hand, the colloids can also promote the heterogeneous nucleation step of the water crystallization kinetics that takes place inside the SSHE, resulting in a decreasing of supercooling and therefore in an increasing in the initial freezing point. As suggested by the experimental cooling curves, both the NUR and IS parameters increased while HNT parameter decreased in the milk-based model systems with respect to the water-based model systems. Under our experimental conditions, the balancing between the two effect resulted in lower levels of the freezing point depression and therefore in higher values of the initial freezing point for the milk-based model systems with respect to the water-based ones. Despite they have very close levels (i.e., about 1.80) of Mw/Ms, the WSUCR + WSORB samples showed an average value of freezing point depression (IFP at −1.58 °C) lower than that (IFP at −2.35 °C) of the MSUCR ones. Similar results were observed by comparing MSUCR + MDEXTR against WSORB samples for which Mw/Ms was close to 1.38: the sucrose equivalents were 51.85 and 55.99 g/100 g of water while the initial freezing point were −2.94 and −2.12 °C for MSUCR + MDEXTR and WSORB, respectively.

An intermediate strength of the colligative solute-solvent interaction was observed in the ice cream mixtures. Such behavior was attributed to the main technological functions of stabilizers and emulsifiers from the multicomponent basis used in their formulations. Concerning the stabilizers, carrageenan, Tara-seed flour and carboxymethylcellulose are the hydrocolloids with the greatest water binding capacity and thickening ability, followed by inulin and maltodextrin. Such high molecular weight hydrocolloids have a great ability to interact with free water molecules through hydration (e.g., carboxymethylcellulose) and gelling (e.g., carrageenan), thus occupying a large solution volume and entangling and interacting with each other and with milk proteins and partially-destabilized fat globules to form large hydrophilic colloids [5]. The synergic ability to form hydrogen bonds with free water molecules at a colloidal scale subtracting them from colligative solute interaction in the unfrozen phase was used to justify the stronger effect on the freezing point depression observed in the ice cream mixtures respect to that observed in milk-based model systems. Such results were in agreement with literature: freeze concentration of the unfrozen serum phase results in a polysaccharide concentration several times higher than that was present in the original mix [5]. Nevertheless, in the case of ice cream mixtures, a counterbalancing effect of the finely dispersed air bubbles on the degree of dependence of Mw/Ms and the freezing point depression was hypothesized: the presence of air reduced the slope in ILP. Although MSORB, MDEXTR and HSC samples were respectively characterized with 1.38, 1.10 and 1.04 for Mw/Ms, the freezing point depression was in the following decreasing order MSORB (−3.85 °C), MDEXTR (−3.45 °C) and HSC (−2.87 °C). The sucrose esters present in the multicomponent basis used in the formulation of the ice cream mixtures promote destabilization of the milk fat globules, enhancing the formation of small and finely dispersed air bubbles inside the unfrozen serum phase. The simultaneous presence of emulsifiers and stabilizers makes more stable the air whipping process at a colloidal scale. The stable incorporation of finely disperse air cells surrounded by milk proteins and partially-destabilized fat globules occurring in the HSC samples concurred to the heterogeneous nucleation process by decreasing the supercooling required, so that water crystallization in the HSC samples occurred at equilibrium temperatures higher that those required for water-based model systems. The average value of the HNT parameter was about 0.68 min, that is, intermediate between those of water-based (0.28 min) and milk-based (1.73 min) model systems. 

Compelling evidence of the destabilizing effects of the emulsifying hydrocolloids, resulting in the increase of the interactions on the colloidal scale, is provided in the (Figure 5b) that shows the Weast’s relationship referred either to the model systems obtained by adding an amount of sugars equal to 300 g and to the MSC ice cream mixtures having the same total solid content but obtained by adding an amount of sugars equal to 200 g. Regardless of their lower concentration of sugars, the MSC samples showed the highest strength of colligative solute-solvent interaction (intercept of the Weast’s function was 0.935) but the lowest degree of dependence between the freezing point and Mw/Ms (the slope of the Weast’s function was 1.348).

### 3.5. Modeling of Crystallization Kinetics for Ice Cream Mixtures

The mathematical description of the water crystallization in batch ice cream production was performed through a phenomenological modeling of the overall freezing occurring inside the SSHE that include the main freezing steps observed on a macroscopic scale, that is, the heterogeneous nucleation, ice crystal growth and air incorporation. 

From one hand, the improvement of the quality of ice cream produced in batch SSHE requires accurate and quantitative data about the composition of the starting liquid mixture and its freezing properties but also about the way in which structural and physical changes occurring under dynamic freezing conditions may affect melting and sensory properties of the final ice cream. From another hand, the time-temperature history experienced by the ice cream mixture inside the SSHE can be used as an accurate fingerprinting of the multiple events taking place on a wide range of scale size and that include heterogeneous nucleation, ice crystal growth, destabilizing of fat globules, cross-linking of hydrocolloids to form a viscoelastic network and air incorporation. 

In order to characterize the water crystallization kinetics that takes place inside the SSHE during ice cream production, the mass fraction of the initial water content was used as a kinetic descriptor of the temperatures experienced by the ice cream mixtures inside the SSHE. On the other hand, the direct measurement of the freezing point depression of the ice cream mixture under dynamic conditions is difficult to obtain instrumentally as a consequence of (i) the increased sample viscosity which is responsible of the slowdown of the rate of heat transfer and (ii) the dynamic scraping conditions causing the ice nuclei to be sheared, melted and mixed on the large ice crystals.

The initial freezing point IFP and freezable water fraction WF(t) are the two parameters required to determine the temperature dependence of the amount of ice that can be separated inside the SSHE during cooling. Other studies provided evidence that the freezable water fraction increases with an increase in the molecular weight of solute and that the temperature dependence of the fraction of frozen water is different between solutions with low molecular and high molecular solutes reflecting the difference in their hydration states [26]. It is widely recognized that the most accurate method to measure the initial freeing point inside the SSHE is based on the analysis of the temperature profile as registered in-situ. The initial freezing point was experimentally determined by performing the first derivative analysis of the cooling curve [27]. They recognized the initial freezing point as the first temperature at which the rate of cooling undergoes a significant decrease because of the supercooling event. A pure solution under freezing shows a decreasing in the minimum when supercooling takes place and that temperature increases to reach an upper plateau (with zero-slope) where the ice crystals begins to growth. The highest temperature level reached after supercooling represents the freezing point for that solution. However, the temperature domain with zero slope is not easy to detect in the case of multicomponent solutions. 

In the present work, the freezable water fraction was based on the numerical analysis of the whole cooling curve throughout three preliminary steps that include (i) the individuation of the first supercooling event, (ii) the determination of IFP and (iii) the calibration of the whole cooling curve. Finally, a re-parameterized Gompertz’s function was used to describe the water crystallization kinetics by quantifying the time-dependent changes in WF(t). 

**Figure 6:**
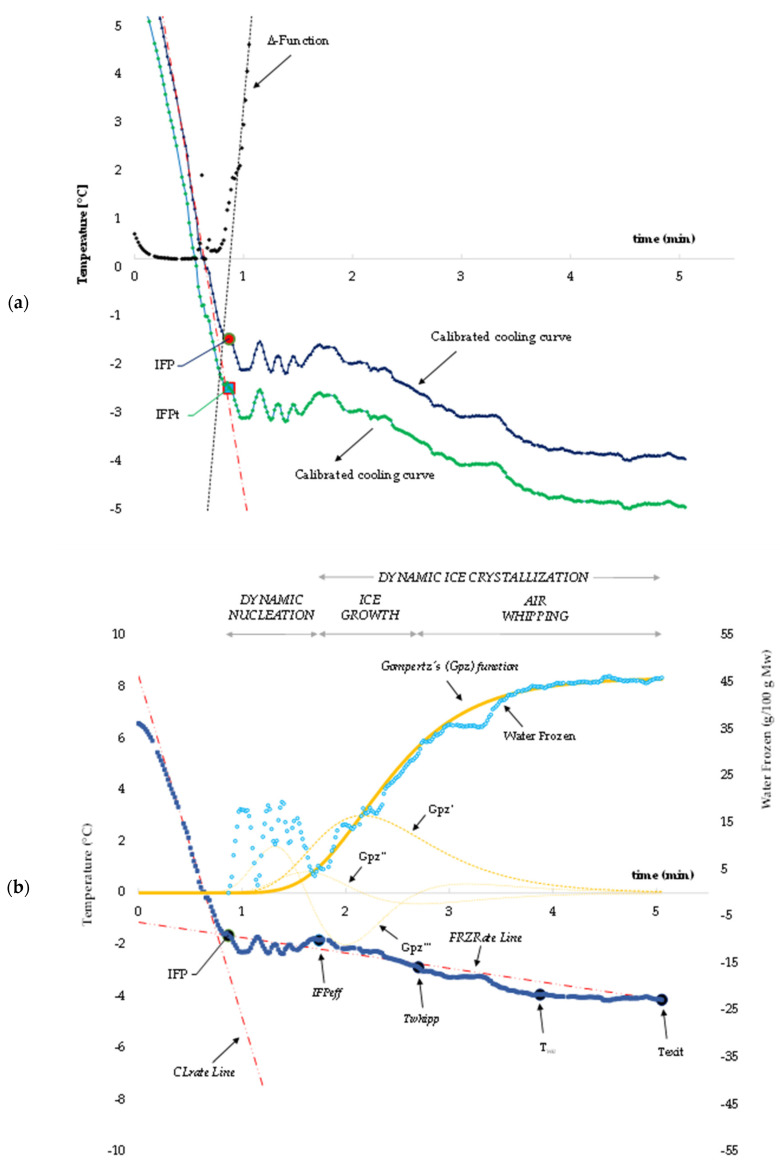
Modeling of Crystallization Kinetics of the HSC ice cream mixture: (**a**) cooling curve calibration; (**b**) Gompertz’s model of the ice crystallization kinetics.

As an example, (Figure 6a) shows the three steps focused on the individuation of the initial freezing point and calibration of the cooling curve for the HSC mixture; while (Figure 6b) shows the superimposition of WF(t) values and those calculated by using the a re-parameterized Gompertz’s function.

In the first step, derivative analysis of the cooling curve was performed to evaluate the time corresponding to the first supercooling event according to [27]. In the second step, IFP was numerically individuated as the highest temperature after the first supercooling. Aiming to develop a robust algorithm able to determine automatically the first supercooling event on the cooling curve, the following ∆-function was defined and analyzed:

(3) $$\frac{{\left[T_{\mathrm{CL}}\left(t)-(t\right)\right]}^{2}}{T_{\exp}\;(t)}\;\leq \;10$$

The value of the function is expected to rapidly increase at the first supercooling (since the ice nucleation is an exothermal process) before the rate of the increasing can reach an asymptotical level during freezing (Figure 6a). The quadratic form of the function allowed to minimize the local dynamic changes of the experimental temperatures. Finally, the time corresponding to the IFP temperature was individuated by determining the time-intercept of the regression line passing through at least ten among the highest values estimated using the ∆-function. 

In the present work, the freezable water fraction  WF(t) was estimated according to the well-established sucrose equivalent method [5] as the mass fraction of the initial water that can be virtually converted into the ice at a given equilibrium freezing temperature, by assuming that the ice cream mixture would behave as a pure sucrose solution. Such an indirect methodology allowed the estimation of this mass fraction in the temperature domain ranging between IFP and T_exit_, which was assumed to include all the equilibrium freezing temperatures experienced by the ice cream mixture inside the freezer. To this aim, the whole cooling curve was previously calibrated by algebraically subtracting the difference calculated between the experimental value of the initial freezing point IFP and the theoretical value IFPt. The calibration of the experimental temperature profile was necessary to compensate the overestimation of the temperature levels measured by the temperature probe due to the specific location inside the SSHE, that is, at intermediate position between the colder zone (freezing surface) and warmer zone (shaft rotor) and the effects exerted by other experimental factors that include the time-of-response of the temperature probe and the solute-solute and solute-solvent interactions. 

During freezing, the concentration of the colligative solutes increases in the unfrozen serum phase, thus causing the progressive reduction of the equilibrium freezing temperature.

In the present work, the temperature dependence of the freezable water fraction was established by coupling the $T_{\exp}\;\left(t\right)$ data to the reverse empirical relationship between the FPD values tabulated for pure sucrose solutions with different sucrose concentrations and the corresponding WF(t). WF(t) was calculated by simulating a 14-point level dataset for the pure sucrose solutions with each level corresponded to a 5% increase, supposing that not over 70% of the initial water can be converted in the ice (such condition must be taken into account to consider as valid the relationship between IFPt and the concentration of sucrose equivalents [SE] in a pure sucrose solution). A fourth-order polynomial function was fitted to the simulated FPD-WF(t) data. 

The assumption that the bulk temperatures of the ice cream mixture correspond to its equilibrium freezing points is supported by several basic studies focused on sugar solutions which proved that the bulk temperature inside the SSHE is very close to the thermodynamic equilibrium temperatures (freezing point) since the secondary nucleation rate of ice is fast [5,28]. The assumption that the sucrose equivalent method can be successfully used to estimate the mass fraction of water that can be converted in the ice is supported by pioneering studies [24] on 110 mixes formulated to include a wide variety of ingredients and concentrations. In a comparison between the sucrose equivalent method and the measurements with an osmometer, the authors found that the theoretical approach underestimated or overestimated the initial freezing point of about 0.18 °C, depending on the choice of ingredients.

From a macroscopic point of view, the water crystallization kinetics occurring in the batch SSHE follows a sigmoidal trend as described by the time-dependent changes in the freezable water fraction during cooling. As can be inferred from (Figure 6b), the dynamic nucleation occurring within the IFP-IFP_eff_ range can be considered an induction step of the whole water crystallization process. In this induction step, cooperative phenomena take place on a microscopic scale, which include heat and mass transfer, supercooling, crystallization and melting events, before the ice started to growth significantly at the effective freezing temperature point IFP_eff_. After this equilibrium temperature, the growth of the ice crystals dominates on the heterogeneous nucleation: the growth rate of WF(t) reaches a maximum level before to decelerate and level off to an upper asymptotic level (on a macroscopic scale) towards the T_whipp_ point, where the air incorporation dominates on the ice formation until the T_exit_ point.

A re-parameterized Gompertz’s function was proposed as a phenomenological model to mathematically describe the time-changes in the freezable water fraction WF(t) under the SSHE dynamic conditions:

(4) $${\mathrm{WF}(t)\;=\;\mathrm{WF}}_{\max}\;\times \;\exp\left\{-\exp\left\{\left(\frac{{\mathrm{GR}}_{\max}\;\times \;2.718}{{\mathrm{WF}}_{\max}}\right)\cdot \left(\mathrm{HNT}-t\right)\;+\;1\right\}\right\}$$

The three model parameters were estimated by performing a non-linear fitting procedure of the re-parameterized Gompertz’s function. The lack-of-fit was evaluated by calculating the square relative mean error (E%, always less than 2%):

(5) $$\tilde{E}\;\%\;=\;\frac{1}{n}\;\times \;\sqrt{\frac{{\left[{\mathrm{WF}(t)-\mathrm{WF}(t)}_{\exp}\right]}^{2}}{\mathrm{WF}(t)}}$$

First-, second- and third-order derivative analysis of the modified Gompertz’s function was performed to determine the time-temperature onsets corresponding to the water crystallization kinetics (1) at its maximum acceleration (IFP_eff_), (2) at its maximum rate (GR_time_ and GRT) and (3) at its maximum deceleration (tT_whipp_ and T_whipp_).

The T_whipp_ could be treated as a key freezing parameter useful to decide the optimum time length required to incorporate an enough amount of the air during freezing and therefore to decide objectively the shut-off of the refrigeration system. High-shear rates due to the high-speed of rotating blades inside the SSHE together with the decrease in temperature promote and sustain the formation of a viscoelastic structure in the unfrozen serum phase through milk fat destabilizing, fat coalescing, protein hydration, hydrocolloid interaction and air incorporation. An optimal strength of such viscoelastic structure is required to keep the maximum overrun: the amount of the incorporated air reaches a maximum and then decreases as a balance between the rheological, interfacial properties and mechanical stress established inside the freezing mixture [9,10,17,18].

It is useful to point out that the temperature of the product undergoes intermittent rising and lowering when prolonging the freezing time (tT_exit_) beyond the set point of desired stiffness, due to the fine-modulation of the stiffness target by the electronic system of the SSHE: the smaller crystals melt causing the increase of the bigger ones [5,28], negatively affecting the ice cream texture.

### 3.6. Modeling of Ice Cream Melting Kinetics

While freezing is required for the development of the ice cream structure, melting occurring when the product is exposed to temperatures higher than the freezing ones is responsible for its loss. On a macroscopic scale, melting can be considered a hysteretic reverse process with respect to the ice crystallization one. Previous research [29,30] highlighted a sigmoidal trend for the ice cream melting, expressed as the mass of the drip loss (m_D_) divided by the total mass of the ice cream (m_0_) and plotted against time, as a result of cooperative mechanisms occurring on microscopic scale including heat and mass transfer, ice melting and air escape. Melting was modeled using the Gompertz’s function proposed to model ice crystallization opportunely adapted:

(6) $$\frac{m_{D}}{m_{0}}=\;\exp\left\{-\exp\left[1\;+\;2.718\;\times \;\frac{{\mathrm{LAG}}_{\mathrm{TIME}}-t}{M_{\mathrm{rate}}-1}\right]\right\})$$

The goodness-of-fit was evaluated as calculating the square relative mean error. 

### 3.7. Liking Degree and Sensory Profile Intensity

**Figure 7:**
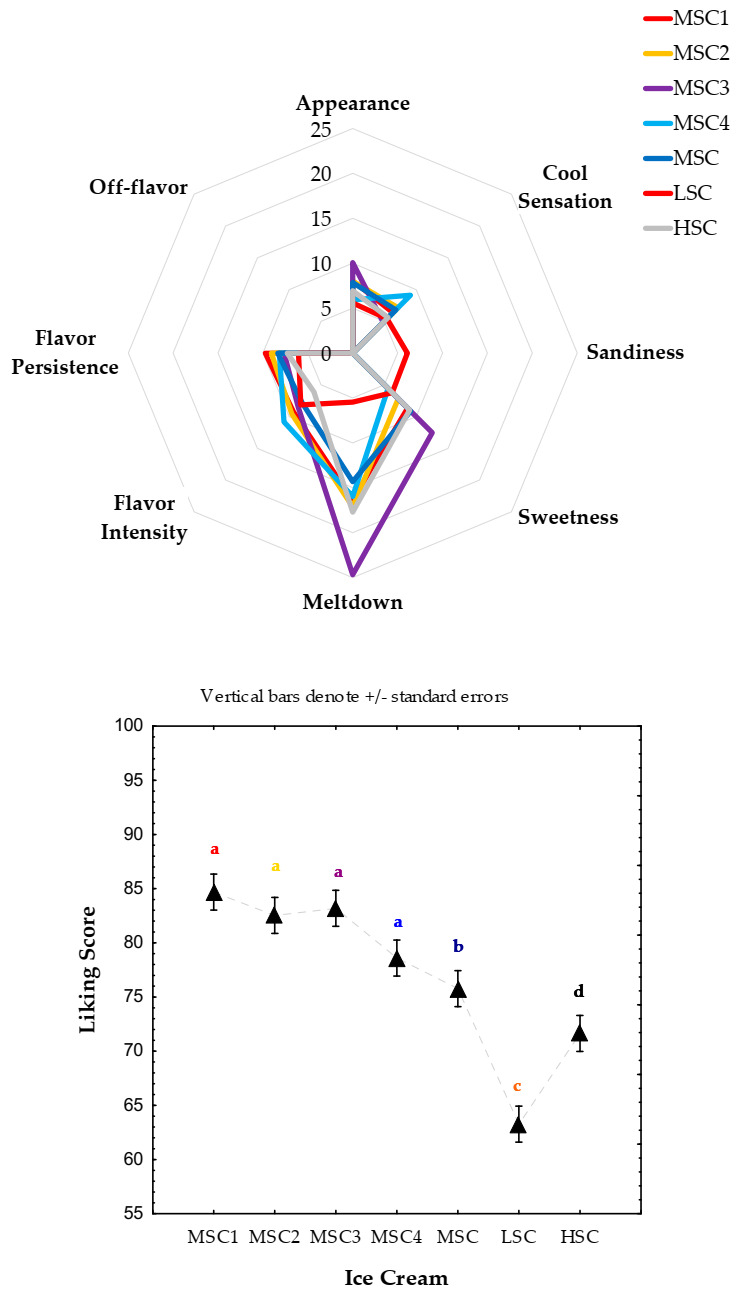
Liking degree and sensory attribute intensity profile for ice cream.

The overall liking of the investigated ice creams was reported in (Figure 7) together with the corresponding attribute intensity profiles. 

As can be inferred from the liking degree, all MSCs were recognized as the “highest-quality ice creams”, LSC the “lowest-quality ice creams” and HSC as the ice creams with intermediate quality. According to the attribute intensity profiles, MSC ice creams were considered the closest to optimal score: MSC1 and MSC2 ice creams mainly for their flavor persistency; MSC4 for their flavor intensity; MSC4 for their cool sensation; MSC3 for their meltdown; and MSC3 for their sweetness. The lowest liking score characterizing the LSC samples was attributed to the sub-optimal perception of all the appreciated sensory attributes and to their high sandiness. However, LSC ice creams were also characterized by the lowest overrun levels (data not showed). Sandiness perception in LSC ice cream was attributed to their highest level of the mass fraction of water converted in the ice (FW) but the presence of lactose crystals was not excluded. It is long term accepted that lactose may transit from metastable to labile state inside an ice cream mainly when the temperature reaches −6.10 °C or −5.6 °C. In our study, LSC ice creams gone out the SSHE with an average bulk temperature close to −3.44 °C. Moreover multiple-agglomeration events registered in the early steps of cooling were also considered a serious factor promoting lactose crystallization.

### 3.8. Relationships between Technological Quality of the Ice Cream Mixture and Ice Cream Quality

Two statistical approaches, namely principal component analysis (PCA) and multiple regression analysis (MRA, were followed in order to find the relationships between the technological quality of the ice cream mixtures and the ice cream quality. In both the cases, composition and freezing properties of the ice cream mixtures were treated as descriptors of their technological quality, while frozen water, overrun, melting rate and sensory properties of the ice cream were treated as final quality parameters. 

**Figure 8:**
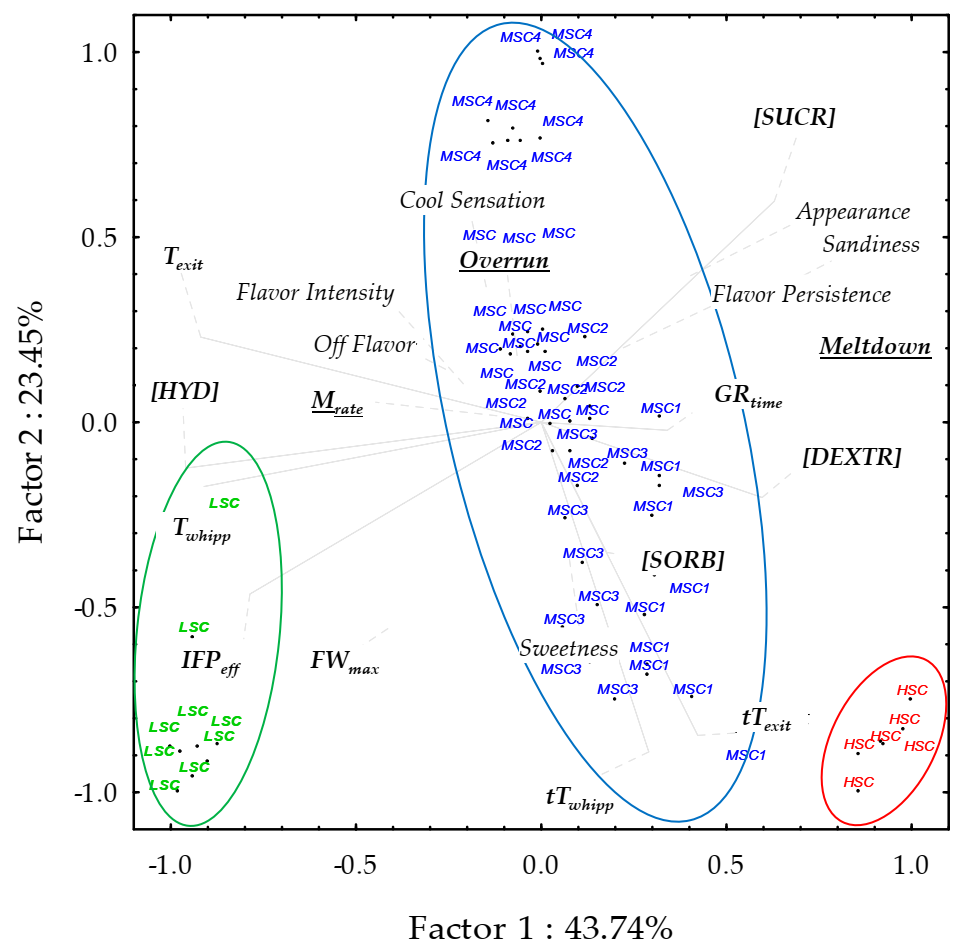
Principal Component Analysis: projection of ice cream samples and variables on the plane.

PCA was firstly performed to highlight the main differences among the ice cream samples as a function of the technological descriptors of the ice cream mixtures and results were interpreted in terms of the ice cream quality. PCA also allowed to select the most significant kinetic properties able to classify the ice creams according to their quality level as described by the overrun, melting and degree of liking. Results were also interpreted in terms of the sensory perception of the ice cream quality. Since ice cream quality is positively correlated to the amount of ice crystals and negatively correlated to their average size, the maximum level of freezable water fraction (FW_max_), together with the time and temperature at the exit point, were also considered indirect indices of both the ice parameters in the final product. These parameters can be compared among the ice cream samples because they were extracted with the same final stiffness. Composition and freezing properties of the ice cream mixtures were used as active variables, while FW_max_, melting rate, overrun and sensory properties of the ice cream were considered supplementary variables and were used only for interpretation purposes. Results from PCA allowed to group the ice cream samples in four clusters of homogeneous samples in the vector space of the original variables as showed in (Figure 8). 

As can be inferred from data, the first two factors accounted for 67.19% of the experimental variance, while the first three factors (data not showed) explained about 83% of it. Although the ice creams were produced with the same final stiffness, they were characterized with a wide range of frozen water, overrun, melting rate values as well as of sensory attributes. The active variables [HYD] (sum of maltodextrin, inulin and other solids), T_whipp_, IFP, T_exit_ were opposite to [SUCR], [DEXTR], [SORB], GR_time_, tT_exit_ and tT_whipp_. These results were coherent with the inverse relationship existing between time and temperature required to reach the same final stiffness ([5] Ross, 1995) and with the structure forming capacity of the hydrocolloids (the higher the hydrocolloid concentration, the shorter the freezing time). The active variables [SUCR], [DEXTR], [SORB], GR_time_, tT_exit_ and tT_whipp_, reported in a decreasing order of factor loading, were positively correlated with appreciated sensory attributes such as a meltdown, smoothness, appearance and flavor persistency and negatively correlated with undesirable factors such as cool sensation and off-flavor. These results could be explained by the interaction of destabilized fat, proteins, emulsifier and structure forming hydrocolloids: they are involved in the development of the unfrozen phase of the ice cream with a structure able to retain flavor and slow the product meltdown. Furthermore, FW_max_ was negatively correlated with appreciated sensory attributes: this is because a high amount of ice is responsible for cold and anesthetic sensation that depress the perception of other sensations. M_rate_ was positively correlated with FW_max_, due to the lower heat capacity of ice with respect to that of liquid water and negatively correlated with Meltdown. This apparent inconsistency could be attributed to the predominant effect of the mechanical shearing of the ice cream between the tongue and palate. FW_max_ was positively correlated with T_exit_, T_whipp and_ IFP as a result of the opposite effect of time and temperature on the ice growth.

As suggested by the projections of the samples on the factor plane (Figure 8), the MSCs ice cream mixtures were characterized by the lowest values of frozen water and melting rate and by the best balance between appreciated and undesirable sensory attributes. The highest levels of IFP, T_exit_, T_whipp,_ FW_max_, tT_whipp_ and tT_exit_ were registered during freezing of the LSC ice cream mixtures, causing the production of ice creams with the lowest-quality. In fact, they were characterized by the highest values of off-flavor, cool sensation, frozen water _and_ meting rate and by the lowest scores for meltdown, flavor persistence, appearance and overrun. HSC ice creams showed intermediate quality between MSC and LSC. 

It is widely known that the greatest number of ice crystals formed in the mechanical freezer are obtained with the highest level of the initial freezing point of the mix, thus resulting in a smoother texture for the ice cream. The initial freezing point also determines the amount of water melts at room temperature. In general, the higher is the initial freezing point, the lowest is the melting rate at room temperature. However, in this study, an opposite behavior was observed for LSC ice cream mixtures for which a prolonged time of freezing was required to incorporate the air and obtain a given overall stiffness at the exit of the SSHE. This unexpected behavior was attributed to an excess of the mechanical energy provided by the SSHE scraping blades during the prolonged freezing time (as indicated by higher levels of tT_whipp_ and tT_exit_), which was able to cause the overrun loss and more likely melting of the smaller ice crystals and growth of the larger ones.

Results from PCA corroborates the idea that some freezing properties obtained from the modeling of the cooling curve can be used for classification purposes of the ice cream mixtures.

Table 11 lists the multiple regression models able to predict some ice cream quality indices together with the results from cross-validation analysis. Composition and freezing parameters were used as input predictors. 

To represent the structural changes taking place during the chilling step of cooling and those occurring during the asymptotic ice formation, CL_rate_ and T_we_ were also considered. The stepwise analysis retained all temperature- and time-domain freezing properties in the predictive models, while all composition properties were discarded from the models, except for the fat concentration that affected the Flavor Intensity attribute. As can be inferred from data, the predictive models were satisfactorily fitted to the calibration data as suggested by the multiple R^2^. The cross-validation of the predictive models provided acceptable results for most ice cream quality indices with exception of those referred to overrun, cool sensation, smoothness and sweetness, whose multiple R^2^ were lower than 0.500.

## 4. Conclusions

A preliminary comparative study of the freezing behavior of water and milk-based model systems and ice cream mixtures was useful to individuate and understand the main freezing steps of the water crystallization kinetics and to highlight the strength of the interactions occurring between the colligative solutes and free water molecules in the SSHE at the conditions required for the batch ice cream production. During the cooling of the ice cream mixtures, more than one supercooling events occurred with lower temperature fluctuations than in the two model systems. This is a consequence of the presence of stabilizers and emulsifiers, which are responsible respectively for the increase of the bulk viscosity and for the extent of the destabilization of the milk fat globules. The whole cooling curve was numerically analyzed and the derived parameters used as descriptors of the main freezing processes underlying the conversion of the colloidal structure of the ice cream mixture into the soft-solid ice cream. 

For the first time, the initial freezing point was numerically individuated *in-situ* starting from the experimental cooling curve registered inside the SSHE and it was identified as the point corresponding to the highest temperature reached after the first supercooling event. A re-parameterized Gompertz’s function was advantageously used to mathematically describe the water crystallization kinetics together with some key kinetic properties. Time-temperature onsets discriminating heterogeneous nucleation, ice growth and air incorporation were objectively determined by performing the first and second-order derivative analysis of the re-parameterized Gompertz’s model. Composition and freezing properties were treated as technological properties of the ice cream mixtures and were used as input variables in the multivariate analysis to classify the ice cream mixtures as a function of their ability to produce high-quality ice creams. Statistical models were developed and validated to predict the final overrun, ice cream melting rate and some sensory attributes such as appearance, cool sensation, sweetness, meltdown, flavor intensity and flavor persistence.

The proposed methodology represents a powerful and science-based approach overcoming the drawbacks of the usual methods based on trial-and-error assessment for setting ice cream formulations and freezing time. The numerical analysis of the experimental time-temperature profiles acquired under dynamic freezing conditions could provide an affordable decision-tool to opportunely select the starting ice cream mixture formulations able to obtain the desired quality properties in the final product. It also represents the first step to build robust predictive models of the ice cream quality indices to be transferred at the industrial level with the perspective to develop a computer-aided tool able to support the managing of the freezing time during batch ice cream production. Obviously, the successive step is to collect a large data set of cooling data accounting for a greater number of ice cream ingredients and their combinations.

## Figures and Tables

**Table 1:** Weight (g) of the ingredients in the model system.

	WSUCR	WSUCR + WSORB	WDEXTR	WSORB	MSUCR	MSUCR + MDEXTR	MDEXTR	MSORB
Added Sugars	300	300	300	300	300	300	300	300
Sucrose	300	150	0	0	300	150	0	0
Dextrose	0	0	300	0	0	150	300	0
Sorbitol	0	150	0	300	0	0	0	300
Water	1000	1000	1000	1000	0	0	0	0
Whole Milk	0	0	0	0	1000	1000	1000	1000
Model system	1300	1300	1300	1300	1300	1300	1300	1300

**Table 2:** Mass balance (g/100 g) of the model systems.

	WSUCR	WSUCR + WSORB	WDEXTR	WSORB	MSUCR	MSUCR + MDEXTR	MDEXTR	MSORB
Sugars	22.97	22.94	21.69	22.96	26.62	25.98	25.35	26.61
Fats	0.00	0.00	0.00	0.00	1.19	1.19	1.19	1.19
MSNF	0.00	0.00	0.00	0.00	2.56	2.55	2.55	2.55
NaCl	0.00	0.00	0.00	0.00	0.10	0.10	0.10	0.10
Total Solids (TS)	22.97	22.94	21.69	22.96	30.47	29.83	29.19	30.46
Total Water (Mw)	77.03	77.06	78.31	77.04	69.53	70.17	70.81	69.54

**Table 3:** Weight (g) of the ingredients of the ice cream mixtures.

	LSC	HSC	MSC	MSC1	MSC2	MSC3	MSC4
Total Added Sugars	100	300	200	200	200	200	200
Sucrose	50	150	150	125	100	100	150
Dextrose	0	50	50	50	50	25	0
Sorbitol	50	100	0	25	50	75	50
Whole Milk	1000	1000	1000	1000	1000	1000	1000
Multicomponent basis	100	100	100	100	100	100	100
Ice cream mixture	1200	1400	1300	1300	1300	1300	1300

**Table 4:** Mass balance (g/100 g) of the ice cream mixtures.

	LSC	HSC	MSC	MSC1	MSC2	MSC3	MSC4
Sugars	18.34	29.74	24.37	24.37	24.37	24.47	24.58
Fats	1.32	1.13	1.22	1.22	1.22	1.22	1.22
MSNF	4.20	3.60	3.88	3.88	3.88	3.88	3.88
NaCl	0.14	0.12	0.13	0.13	0.13	0.13	0.13
Total Solids (TS)	23.99	34.58	29.59	29.59	29.59	29.69	29.80
Total Water (Mw)	76.01	65.42	70.41	70.41	70.41	70.31	70.20

**Table 5:** Sucrose equivalents from the colligative sugars of the model systems (SE, g/100 g of water).

	WSUCR	WSUCR + WSORB	WDEXTR	WSORB	MSUCR	MSUCR + MDEXTR	MDEXTR	MSORB
SE from Sucrose	29.81	14.90	0.00	0.06	33.02	16.36	0.00	0.07
SE from Dextrose ^1^	0.01	0.09	52.63	0.17	0.01	29.37	58.21	0.19
SE from Sorbito ^l^	0.00	27.87	0.00	55.75	0.00	0.00	0.00	61.76
SE from Lactose ^2^	0.00	0.00	0.00	0.00	5.26	5.21	5.16	5.25
SE from Fructose ^3^	0.01	0.00	0.00	0.00	0.01	0.00	0.00	0.00
SE from all Sugars	29.82	42.86	52.63	55.98	38.29	50.94	63.37	67.27

^1^ dextrose deriving from both the added dextrose and, in a small amount, the sucrose ingredient; ^2^ lactose deriving from the whole milk; ^3^ fructose contained in a small amount in the sucrose ingredient.

**Table 6:** Sucrose equivalents from colligative sugars in ice cream mixtures (SE, g/100 g of water).

	LSC	HSC	MSC	MSC1	MSC2	MSC3	MSC4
SE from Sucrose	5.55	16.41	16.39	13.68	10.97	10.99	16.45
SE from Dextrose ^1^	6.30	16.06	16.01	16.02	16.04	11.19	6.30
Se form Sorbito ^l^	10.20	20.32	0.00	5.08	10.17	15.27	10.20
SE from Lactose ^2^	6.94	6.91	6.91	6.91	6.91	6.92	6.93
SE from Fructose ^3^	0.03	0.04	0.04	0.04	0.04	0.04	0.04
SE from Maltose ^4^	0.10	0.10	0.10	0.10	0.10	0.10	0.10
SE from Maltotriose ^4^	0.17	0.17	0.17	0.17	0.17	0.17	0.17
SE from Inulin ^4^	0.18	0.17	0.17	0.17	0.17	0.18	0.18
SE from Maltodextrins ^4^	0.17	0.17	0.17	0.17	0.17	0.17	0.17
SE from all Sugars	29.64	60.35	39.96	42.35	44.73	45.03	40.54

^1^ deriving from the added dextrose and, in small amounts, from the multicomponent basis and the sucrose ingredient; ^2^ deriving from the whole milk and from the multicomponent basis; ^3^ deriving in small amounts from the multicomponent basis and the sucrose ingredient; ^4^ deriving from the multicomponent basis.

**Table 7:** List of the sensory attributes and of the corresponding evaluation scales.

Attribute	Score Scale	Description
Appearance	0–5	Visual perception of the ability to retain the initial waves after shaping with a cup without undergo fast surface melting (score = 0 for “doesn’t appeal”; score = 5 for “very appealing”)
Sweetness	0–10	In-mouth sweetness perception (score = 0 for “too low” or “too high” and score = 10 for “optimal level”)
Meltdown	0–10	The time required by the product to melt in the mouth when continuously pressed by the tongue against the palate (score = 0 for “too low” or “too high” and score = 10 for “optimal level”)
Sandiness	0–5	In-mouth textural sensation of the presence of fine, hard, uniform, sand-like particles. It was evaluated by pressing a thin layer of ice cream against the palate of the mouth with the tongue to obtain a quick melting (score = 0 for “very sand texture” and score = 5 for “very smooth texture”)
Cool sensation	0–10	In-mouth trigeminal perception of low temperature (score = 0 for “too low” or “too high” and score = 10 for “optimal level”)
Flavor Intensity	0–8	In-mouth intensity of the overall gustative sensations (score = 0 for “too low” or “too high” and score = 8 for “optimal level”)
Flavor persistence	0–8	In-mouth temporal dominance of the overall gustative sensations (score = 0 for “too low” or “too high” and score = 8 for “optimal level”)
Off-flavor	0–5	Evaluated as retro gustative perception of flavor not typical of ice-cream when the ice cream is in mouth or it has just been swallowed (0 for “without flavor defects,” 5 for “very defective flavor”)

**Table 8:** Characteristics of the reference ice creams.

Attribute	Production Specifications Used to Obtain the Four Intensity Levels (*)			
Appearance and Meltdown	Amount and ratio between total emulsifiers (sucrose esters) and stabilizers in the multicomponent basis			
	(34.65 g + 5.35 g);6.47:1	(35.65 g + 4.35 g); 8.19:1	(36.65 g + 3.35 g); 10.94:1	(37.65 g + 2.35 g); 16.02:1
Sweetness	Amount of total added sucrose			
	100 g	150 g	200 g	250 g
Sandiness	Duration of shut down of the power supply of the tempering cabinet			
	30 min	60 min	90 min	120 min
Cool sensation	Drawing temperature from the SSHE			
	−3 °C	−5 °C	−7 °C	−9 °C
Flavor Intensity, Persistency, Off-flavor	Amount and ratio between the whole milk and multicomponent basis			
	(990 g + 110 g); 9:1	(1000 g + 100 g); 10:1	(1010 g + 90 g); 11.22:1	(1020 g + 80 g); 12.75:1

(*) Reference samples given from left to right are in the order of increasing attribute intensity.

**Table 9:** Theoretical Freezing Point of the model systems: contribution of MSNF, NaCl and sugars to the freezing point depression (FPD).

		WSUCR	WSUCR + WSORB	WDEXTR	WSORB	MSUCR	MSUCR + MDEXTR	MDEXTR	MSORB
Contributionto FPD (°C) of:	MSNF ^1^	0.00	0.00	0.00	0.00	0.09	0.09	0.09	0.09
	NaCl ^2^	0.00	0.00	0.00	0.00	0.09	0.08	0.08	0.08
	Sugars ^3^	1.91	2.79	3.47	3.71	2.48	3.35	4.24	4.52
IFPt (°C) of the model systems		−1.91	−2.79	−3.47	−3.71	−2.65	−3.52	−4.41	−4.70

^1^ It was calculated according to the following equation of Goff and Hartel (2013): ${\mathrm{FDP}({}^{\circ}C)}_{\mathrm{MNSF}}=\;2.37\cdot \left(\frac{\left[\mathrm{MSNF}\right]}{\left[\mathrm{Mw}\right]}\right),$ where [Mw] is the initial water concentration (% by weight). ^2^ It was calculated according to the following equation of Potter et al. (1978): ${\mathrm{FDP}({}^{\circ}C)}_{\mathrm{NaCl}}=\;0.581855\cdot \left(100\cdot \frac{\left[\mathrm{NaCl}\right]}{\left[\mathrm{Mw}\right]}\right)+\;0.0034889\cdot {\left(100\cdot \frac{\left[\mathrm{NaCl}\right]}{\left[\mathrm{Mw}\right]}\right)}^{2}+\;0.0004314\cdot {\left(100\cdot \frac{\left[\mathrm{NaCl}\right]}{\left[\mathrm{Mw}\right]}\right)}^{3}$, where [NaCl] is the initial concentration of NaCl (% by weight). ^3^ It was calculated according to the following equation of Goff and Hartel (2013): $\mathrm{FDP}\left({}^{\circ}C\right)\;=\;0.00009\cdot {\left[\mathrm{SE}\right]}^{2}+\;0.0612\cdot [\mathrm{SE}],$ where [SE] is the initial concentration of sucrose equivalents (% by weight) from all sugars.

**Table 10:** Theoretical and Experimental Freezing Point of the ice cream mixtures: contribution of MSNF, NaCl and sugars to the freezing point depression (FPD).

		LSC	HSC	MSC	MSC1	MSC2	MSC3	MSC4
Contribution to FPD (°C) of:	MSNF ^1^	0.13	0.13	0.13	0.13	0.13	0.13	0.13
	NaCl ^2^	0.10	0.10	0.10	0.10	0.10	0.10	0.10
	Sugars ^3^	1.89	4.02	2.59	2.75	2.92	2.94	2.63
IFPt (°C) of ice cream mixtures		−2.13	−4.26	−2.82	−2.99	−3.15	−3.17	−2.86
IFP (°C) of ice cream mixtures		−1.52	−2.87	−1.92	−2.99	−3.15	−3.17	−2.86
∆IFP (°C)		0.61	1.39	0.90	0.89	0.60	0.74	0.47

^1^ It was calculated according to the following equation of Goff and Hartel (2013): ${\mathrm{FDP}({}^{\circ}C)}_{\mathrm{MNSF}}=\;2.37\cdot \left(\frac{\left[\mathrm{MSNF}\right]}{\left[\mathrm{Mw}\right]}\right),$ where [Mw] is the initial water concentration (% by weight). ^2^ It was calculated according to the following equation of Potter et al. (1978): ${\mathrm{FDP}({}^{\circ}C)}_{\mathrm{NaCl}}=\;0.581855\cdot \left(100\cdot \frac{\left[\mathrm{NaCl}\right]}{\left[\mathrm{Mw}\right]}\right)+0.0034889\cdot {\left(100\cdot \frac{\left[\mathrm{NaCl}\right]}{\left[\mathrm{Mw}\right]}\right)}^{2}+\;0.0004314\cdot {\left(100\cdot \frac{\left[\mathrm{NaCl}\right]}{\left[\mathrm{Mw}\right]}\right)}^{3}$, where [NaCl] is the initial concentration of NaCl (% by weight). ^3^ It was calculated according to the following equation of Goff and Hartel (2013): $\mathrm{FDP}\left({}^{\circ}C\right)\;=\;0.00009\cdot {\left[\mathrm{SE}\right]}^{2}+\;0.0612\cdot [\mathrm{SE}],$ where [SE] is the initial concentration of sucrose equivalents (% by weight) from all sugars.

**Table 11:** Application of Stepwise Regression Analysis for the prediction of the ice cream quality indices.

Multiple Regression Models	Multiple *R*^2^	*p*	Calibration Model (70%)	Multiple *R*^2^	*p*	Validation Data (30%)	Multiple *R*^2^	*p*
M_rate_ = −0.61 HNT−1.45·tT_exit_ + 0.12 CL_rate_ + 0.69 GR_time_	0.775	4.4 × 10^−4^	Pred = 0.56 + 0.78·Obs	0.775	0.000	Pred=1.11 + 0.56·Obs	0.526	0.000
Overrun = 21.54 T_we_ + 25.03·IFP−5.82·IFP_eff_-14.33 tT_exit_	0.717	3.9 × 10^−3^	Pred = 8.39 + 0.72·Obs	0.717	0.000	Pred=19.87 + 0.31·Obs	0.421	0.018
Appearance = −2.91·T_we −_ 0.94·HNT	0.817	5.4 × 10^−5^	Pred = 0.61 + 0.82·Obs	0.817	0.000	Pred=0.93 + 0.73·Obs	0.548	0.000
Cool sensation = 5.19 − T_[IFP-2]_ − 3.07 GR_time_ + 2.30·HNT+5.3·tT_exit −_ 7.13 IFP + 0.39·CL_rate_	0.798	1.5 × 10^−4^	Pred = 1.18 + 0.79·Obs	0.798	0.000	Pred=2.18 + 0.62·Obs	0.426	0.000
Smoothness = 0.33 tT_whipp_	0.792	2.1 × 10^−4^	Pred = 1.03 + 0.79·Obs	0.792	0.000	Pred=0.56+ 0.78·Obs	0.401	0.000
Sweetness = 2.65·IFP_eff_ − 2.34·HNT	0.805	1.1 × 10^−4^	Pred = 1.46 + 0.80·Obs	0.805	0.000	Pred=2.22 + 0.69·Obs	0.486	0.000
Meltdown = −3.80·T_we_	0.872	1.2 × 10^−6^	Pred = 0.86 + 0.87·Obs	0.872	0.000	Pred=1.10 + 0.85·Obs	0.672	0.000
Flavor Intensity = −2.31·GR_time_ + 1.13·HNT − 0.24·CLrate + 6.35·FAT	0.865	2.2 × 10^−6^	Pred = 1.04 + 0.87·Obs	0.865	0.000	Pred=2.15 + 0.71F·Obs	0.709	0.000
Flavor Persistence = −1.942·HNT	0.775	0.001	Pred = 1.667 + 0.775·Obs	0.775	0.000	Pred=2.174 + 0.715·Obs	0.523	0.000

## Data Availability

Not applicable.

## Abbreviations

CLrate, Chilling Rate	the Newtonian cooling rate, that is, the rate at which the solution experiences a decrease in temperature without ice nucleation and growth.
[DEXTR]	the initial concentration (% by weight) of the dextrose in the ICM.
[HYD]	the initial concentration (% by weight) of all hydrocolloids in the ICM (i.e., maltodextrin, inulin, sucrose esters, carrageenan, Tara seed flour and carboxymethylcellulose arising from the multicomponent basis used in the formulation of the ice cream mixtures).
[FAT]	the initial concentration of fat in the ICM.
FP, Freezing Point (°C)	the equilibrium freezing temperature experienced by the WBMS, at which the ice crystal starts to growth with a steady-state kinetics (i.e., without changes in temperature): it was determined as the highest temperature after the unique supercooling event as registered by the lowest experimental temperature.
FPD, Freezing Point Depression (°C)	the fall of the equilibrium freezing temperature below 0 °C due to the presence of colligative solutes.
FPt, Theoretical Freezing Point	the theoretical equilibrium freezing temperature that the WBMS can virtually experience on cooling if they would behave as a pure sucrose solution having an equivalent concentration of sucrose.
${\mathrm{GR}}_{\max}$	the maximum rate (1/min) of the water crystallization (or equivalently, the maximum Growth Rate of the ice crystals) that takes place during batch ice cream production.
Gpz’	first derivative function of the re-parameterized Gompertz’s model.
Gpz,”	second derivative function of the re-parameterized Gompertz’s model.
Gpz’’’	third derivative function of the re-parameterized Gompertz’s model.
GR_time_	the freezing time corresponding to GR_max._
GR_T_	the freezing temperature corresponding to GR_max._
HNT, Heterogeneous Nucleation Time	the time length (min) required to form stable ice nuclei through one (as experienced by the WBMS) or more than one (as experienced by the MBMS and ICM) supercooling events before to reach respectively FP or IFP.
HSC, High Sugar Content	ice cream mixture prepared with the highest content of the colligative sugars.
ICM	Ice Cream Mixture.
IFP, Initial Freezing Point (°C)	the first equilibrium freezing temperature experienced during cooling by the investigated MBMS and ICM (it was determined as the highest temperature after the first supercooling event as registered on the experimental cooling curve).
IFP_eff_, Effective Initial Freezing Point (°C)	the equilibrium freezing temperature at which the water crystallization kinetics is at its acceleration point, after the heterogeneous nucleation step and where the ice starts to accumulate significantly and extensively in the ICM (it was estimated by using the re-parameterized Gompertz’ model).
IFPt, Theoretical Freezing Point	the theorical equilibrium freezing temperature that the investigated MBMS and ICM can experience virtually if they would behave on cooling as a pure sucrose solution having an equivalent concentration of sucrose.
ILP	Interaction Linear Plot obtained by linear regression analysis of the experimental data corresponding to the inverse values of the freezing point depression (x-data) and the ratio between Mw/Ms (y-data).
IS, Initial Supercooling (°C)	the maximum temperature depression below 0 °C experienced on cooling by the investigated MBMS and ICM at the point corresponding to the first ice nuclei formation.
LAG_TIME_	the time at which melting of the ice cream accelerates.
LSC, Low Sugar Content	ice cream mixture prepared with the lowest total sugar content.
MBMS	Milk-based model systems (i.e., MSUCR, MSUCR + MDEXTR, MDEXTR, MSORB).
m_0_	the total mass of the ice cream.
m_D_	the mass of the drip loss during melting of the ice cream.
$M_{\mathrm{rate}}$	the maximum rate (1/min) of the ice cream melting.
Ms	the concentration of the sucrose equivalents (expressed as g/100 of water) corresponding to all the colligative solutes.
MSC, MSC1, MSC2, MSC3 and MSC4	Medium Sugar Content: ice cream mixtures prepared with the same intermediate total sugar content but varying the concentration of the individual sugar added as sweeteners, that is, sucrose, dextrose and sorbitol.
MSNF, Milk Solids Non-Fat	they include all solids in milk other than fat in the ICM.
MSUCR, MDEXT, MSORB	milk-based model systems prepared by adding 300 g of sucrose or dextrose or sorbitol to 1000 g of whole milk, respectively.
MSUCR + MDEXTR	milk-based model systems prepared by adding 150 g of sucrose and 150 g of dextrose to 1000 g of whole milk.
Mw	the mass fraction (% by weight) of the initial water of the ICM.
n	the number of time-temperature data registered by the PT100 probe inside the SSHE.
NUR, Nucleation Rate (°C/min)	the rate of formation of unstable ice nuclei in the unique supercooling domain (as experienced by the WBMS) or in the first supercooling domain (as experienced by the MBMS and ICM).
S, Supercooling (°C)	the maximum temperature depression below 0 °C required by the water to begin crystallization in the WBMS.
[SE]	the concentration (% by weight) of the sucrose equivalents as calculated by summing the individual contribute of all colligative solutes present in a given solution, provided that the it would behave as a pure sucrose solution (“sucrose equivalency” was derived for a comparison of the mean molecular weights for each colligative solute with that of the sucrose).
[SORB]	the initial concentration (% by weight) of the sorbitol in the ICM.
SSHE	Scraped-Surface Heat Exchanger used for batch ice cream production.
[SUCR]	the concentration (% by weight) of the sucrose.
$T_{\exp}\left(t\right)$	the experimental temperature (°C) at a given time (t) registered by the PT100 inside the SSHE.
T_CL_ (t)	the theoretical temperature (°C) at a given time (t), that either the MBMS and the ICM would experience under a pure Newtonian cooling process, that is, without experience ice nucleation and ice growth.
T_exit_	the temperature (°C) at which the ice cream exits from the SSHE.
TP1	First target point: the temperature at which the formation of unstable clusters of ice nuclei begins and the cooling curve starts to deviate from its linear trend experienced during the chilling step of cooling: it was estimated by determining the minimum significant difference between the experimental temperature $T_{\exp}\left(t\right)$ and the theoretical temperature TCL (t).
TS, Total Solids	the total mass fraction (% by weight) that includes all solids that are present in the WBMS, MBMS and ICM.
tT_exit_	the time (min) corresponding to T_exit._
tT_whipp_	the time (min) corresponding to T_whipp._
T_we_	the temperature (°C) corresponding to the intermediate time between T_whipp_ and T_exit._
T_whipp_	the temperature (°C) at which the water crystallization kinetics is at its decelerating point and air incorporation starts to dominate on the ice growth (it was estimated by using the re-parameterized Gompertz’ model).
WBMS	Water-based model systems (i.e., WSUCR, WSUCR + WSORB, WDEXTR, WSORB)
$\mathrm{WF}\left(t\right)$, Freezable Water Fraction (%)	the time-dependent changes in mass fraction (% by weight) of the water initially present in the ICM and that can be virtually converted in the ice during freezing under batch SSHE conditions, as estimated by using the re-parameterized Gompertz’s function.
$\mathrm{WF}{\left(t\right)}_{\exp}$, Experimental Freezable Water Fraction	the time-dependent changes in mass fraction (% by weight) of the water initially present in the ICM and that can be virtually converted in the ice during freezing under batch SSHE conditions, as estimated by coupling the experimental time-temperature data to the reverse relationship between the freezing point depression values tabulated for pure sucrose solutions with different sucrose concentrations and the corresponding mass fraction of the initial water that can be converted in the ice.
${\mathrm{WF}}_{\max}$	the upper asymptotic level of $\mathrm{WF}\left(t\right)$.
W_ice cream_	the weight of a standard volume of the just extruded ice cream (used for overrun measurements).
W_mixture_	the weight of the ice cream mixture after aging corresponding to the same volume of W_ice cream_ (used for overrun measurements).
WSUCR, WDEXT, WSORB	water-based model systems prepared by adding 300 g of sucrose or dextrose or sorbitol to 1000 g of water, respectively.
WSUCR + WSORB	water-based model systems prepared by adding 150 g of sucrose and 150 g of sorbitol to 1000 g of water.
∆FP	the difference between the FPt and FP.
